# New Ikarugamycin Derivatives with Antifungal and Antibacterial Properties from *Streptomyces zhaozhouensis*

**DOI:** 10.3390/md13010128

**Published:** 2014-12-29

**Authors:** Rodney Lacret, Daniel Oves-Costales, Cristina Gómez, Caridad Díaz, Mercedes de la Cruz, Ignacio Pérez-Victoria, Francisca Vicente, Olga Genilloud, Fernando Reyes

**Affiliations:** Fundación MEDINA, Centro de Excelencia en Investigación de Medicamentos Innovadores en Andalucía, Avda. del Conocimiento 34, Armilla, Granada 18016, Spain; E-Mails: daniel.oves@medinaandalucia.es (D.O.-C.); cristiga90@gmail.com (C.G.); caridad.diaz@medinaandalucia.es (C.D.); mercedes.delacruz@medinaandalucia.es (M.C.); ignacio.perez-victoria@medinaandalucia.es (I.P.-V.); francisca.vicente@medinaandalucia.es (F.V.); olga.genilloud@medinaandalucia.es (O.G.)

**Keywords:** *Streptomyces zhaozhouensis*, ikarugamycin, polycyclic tetramate macrolactams, antifungal, antibacterial

## Abstract

A bioassay guided fractionation of the ethyl acetate extract from culture broths of the strain *Streptomyces zhaozhouensis* CA-185989 led to the isolation of three new polycyclic tetramic acid macrolactams (**1**–**3**) and four known compounds. All the new compounds were structurally related to the known *Streptomyces* metabolite ikarugamycin (**4**). Their structural elucidation was accomplished using a combination of electrospray-time of flight mass spectrometry (ESI-TOF MS) and 1D and 2D NMR analyses. Compounds **1**–**3** showed antifungal activity against *Aspergillus fumigatus*, *Candida albicans* and antibacterial activity against methicillin-resistant *Staphylococcus aureus* (MRSA).

## 1. Introduction

The ocean has recently been shown to be an ecosystem with many unique forms of actinomycetes [1] whose distribution in the sea is largely unexplored and which have been proven to be a rich source for the discovery of new natural products with therapeutic potential [2,3]. It is well known that obtaining drugs from natural sources is a long and complex process, which has led to international, multidisciplinary projects with the goal of reducing the time between discovery of new biologically active microbial natural products and the development of new pharmaceuticals. One of such projects is the *PharmaSea* consortium, whose goals are the identification, study, and development of new antibacterial, antifungal, anti-inflammatory and neuroprotective compounds from marine microorganisms isolated from extreme environments. As part of this project, over 400 microorganisms, including strains from the extensively studied families *Streptomycetaceae* and *Micromonosporaceae*, as well as other minor taxa such as *Micrococcaceae*, *Intrasporangiaceae*, *Microbacteriaceae*, *Nocardiopsaceae*, *Nocardiaceae* and *Mycobacteriaceae*, were grown on carefully selected fermentation media, and their fermentation extracts were assayed against clinically relevant pathogenic microbial strains: Gram-positive bacteria (methicillin-resistant *S. aureus*, MRSA), Gram-negative bacteria (*Escherichia coli* and *Pseudomonas aeruginosa*), and fungi (*A. fumigatus* and *C. albicans*).

Growth inhibition of *A. fumigatus*, *C. albicans* and MRSA was observed in acetone extracts from fermentation broths of strain CA-185989, which upon 16S rRNA sequencing was found to be closely related to the recently described species *Streptomyces zhaozhouensis* NEAU-LZS-5(T) [4]. A bioassay-guided fractionation of extracts of this microorganism was carried out in order to isolate and identify the chemical constituents that were responsible for the observed activities. We report herein the isolation of three new polycyclic tetramic acid macrolactams (PTMs) (**1**–**3**) and four known compounds (**4**–**7**). Their structural elucidation was accomplished using a combination of spectroscopic techniques, including HRMS and extensive 1D and 2D NMR analyses. Compounds **1**–**3** possess new structures related to ikarugamycin (**4**), a PTM first isolated from *Streptomyces phaeochromogenes* sub-sp. *ikaruganensis* which showed strong antiprotozoal activity [5,6]. PTMs display a distinctive structure comprised of a macrolactam ring, a series of carbocyclic rings and a tetramic acid ring, and many of them possess antifungal properties [7,8,9,10,11].

## 2. Results and Discussion

### 2.1. Isolation and Taxonomy of the Producing Microorganism

**Figure 1:**
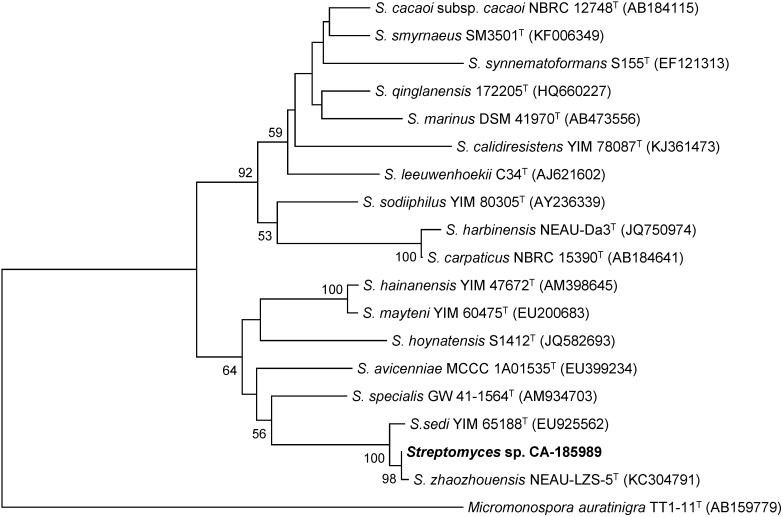
Neighbor-joining tree built with MEGA 6.06 based on nearly-complete 16S rRNA gene sequences of CA-185989 and the closest type strains of the genus *Streptomyces*. *Micromonospora auratinigra* TT1-11(T) was employed as an out-group. The numbers at the nodes indicate bootstrap support (%) based on NJ analysis of 1000 replicates; only values higher that 50% are shown. The scale bar indicates 0.01 substitutions per site.

The producing strain, CA-185989, was isolated from a marine sediment collected off-shore at two meters depth nearby Utonde, Equatorial Guinea. The BLASTN search [12] with the 16S rRNA gene sequence (1379 nt) indicated that the strain is closely related to the recently described *S. zhaozhouensis* NEAU-LZS-5(T) [4]. A phylogenetic tree generated using the neighbor-joining method corrected with the Jukes and Cantor algorithm [13,14] showed the relatedness of strain CA-185989 with *S. zhaozhouensis* NEAU-LZS-5(T) (99.93% sequence similarity) and *Streptomyces sedi* YIM 65188(T) (99.56% sequence similarity), a relatedness highly supported by the bootstrap values (Figure 1). The remaining closest members of the genus *Streptomyces* exhibited sequence similarities below 97.3%.

### 2.2. Bioassay-Guided Isolation

**Figure 2:**
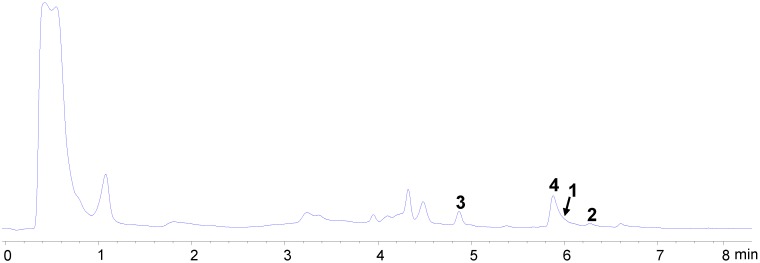
Chromatographic UV trace (210 nm) from the LC-UV-MS analysis of aqueous crude extract (ACE) where compounds **1**–**4** were detected.

The producing strain, *S. zhaozhouensis* CA-185989, was fermented at 28 °C in 1 L of APM9-modified medium during 6 days. Extraction with an equal volume of acetone and evaporation of the solvent afforded an aqueous crude extract (ACE), which was subsequently subjected to liquid-liquid extraction with ethyl acetate. LC-UV-MS analysis of the ACE (Figure 2) revealed the presence of ikarugamycin and some related PTMs which were not included in our internal microbial natural products library [15,16,17] and whose molecular formulae suggested the presence in the extract of new natural products. The resulting aqueous and organic extracts were assayed against *A. fumigatus* and *C. albicans*, with the organic extract being the most active one. This extract was chromatographed on silica gel using hexane/ethyl acetate mixtures of increasing polarity.

**Figure 3:**
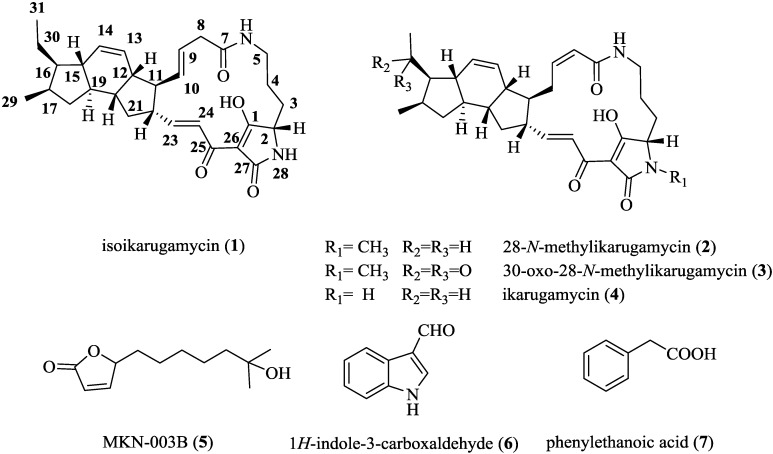
Compounds isolated from culture broths of of *Streptomyces zhaozhouensis*.

Further chromatographic separation of bioactive fractions from this chromatography on a reversed phase C8 column using a gradient CH_3_CN/H_2_O with 0.1% trifluoroacetic acid, allowed us to isolate seven compounds (**1**–**7**) (Figure 3). The spectroscopic data of **4**–**7** were identical to those previously reported for ikarugamycin (**4**) [5,6,18], MKN-003B (**5**) [19], 1*H*-indole-3-carboxaldehyde (**6**) [20] and phenylethanoic acid (**7**) [21]. All these compounds were isolated for the first time from the fermentation broths of *S. zhaozhouensis*. On the other hand, compounds **1**–**3** represent new natural products based on the structure of ikarugamycin (**4**).

### 2.3. Structural Determination of the Compounds Isolated

**Figure 4:**
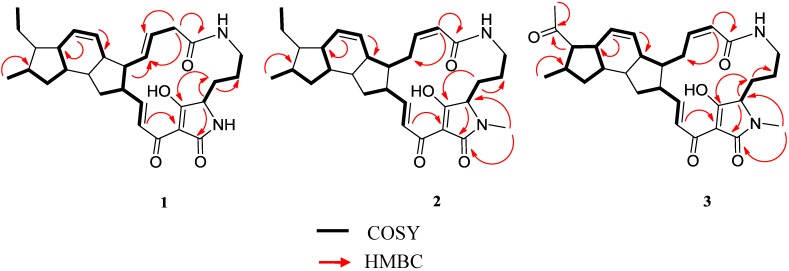
Key COSY and HMBC correlations observed in the spectra of compounds **1**–**3**.

Compound **1** was isolated as a white amorphous solid. The ESI-TOF spectrum of this compound displayed a pseudomolecular ion at *m*/*z* 479.2899 (calc. for [M + H]^+^ 479.2904) corresponding to a molecular formula of C_29_H_38_N_2_O_4_ and accounting for 12 degrees of unsaturation. The ^1^H NMR spectrum (Table 1 and Figure S1) showed the presence of six olefinic protons at δ_H_ 7.04 (1H, d, *J* = 15.7 Hz, H-24), 6.84 (1H, dd, *J* = 15.7, 9.0 Hz, H-23), 5.88 (1H, d, *J* = 10.0 Hz, H-14), 5.55 (1H, d, *J* = 10.0 Hz, H-13), 5.49 (1H, dd, *J* = 14.8, 9.4 Hz, H-9), 5.34 (1H, dd, *J* = 14.8 Hz, 9.4 Hz, H-10) and 5.83 (1H, d, *J* = 10.0 Hz, H-8). On the other hand, two methyl groups in the aliphatic region at δ 0.88 (3H, d, *J* = 7.2 Hz, H-29) and 0.93 (3H, t, *J* = 7.2 Hz, H-31) could also be distinguished. The ^13^C NMR spectrum exhibited 29 signals and the multiplicity edited HSQC spectra suggested the presence of 5 quaternary, 15 methine, 7 methylene and 2 methyl carbons. The spectroscopic data indicated that compound **1** should be a polycyclic tetramic acid macrolactam with ikarugamycin skeleton and the molecular formula further suggested that this compound was an isomer of ikarugamycin (**4**) [18]. The major differences observed between the spectroscopic data of compound **1** and **4** were in agreement with the isomerization of one of the double bonds from the ∆^8^ to the ∆^9^ position. The *E* configuration of the C-9/C-10 double bond was deduced from the coupling constant value of 14.8 Hz between H-9 and H-10. The HMBC experiment confirmed the position of double bond at ∆^9^, with correlations observed between H-8a (δ 2.92) and H-8b (δ 3.05) with C-7 (δ 171.5), C-9 (δ 127.7) and C-10 (δ 133.9), as well as between H-11 (δ 1.88) and C-10 (δ 133.9), as shown in Figure 4.

**Table 1:** ^1^H and ^13^C NMR (500 and 125 MHz in CDCl_3_) data for compounds **1**, **2** and **3**.

Position	Isoikarugamycin (1)		28-*N*-Methylikarugamycin (2)		30-Oxo-28-*N*-Methyl Ikarugamycin (3)		
	δ H, Mult., (*J* Hz)	δ C, Mult.	δ H, Mult., (*J* Hz)	δ C, Mult.	δ H, Mult., (*J* Hz)		δ C, Mult.
1		196.7, C		195.3, C			195.2, C
2	3.95, br s	61.4, CH	3.72, d (3.5)	66.2, CH	3.71, d (4.0)		66.3, CH
3	1.92, m2.02, m	27.0, CH_2_	1.79, d (15.3) 2.14 m	25.0, CH_2_	1.79, d (15.8) 2.13 m		24.9, CH_2_
4	1.10, m1.67, m	22.3, CH_2_	1.13, m 1.47, m	20.6, CH_2_	1.10, m 1.47 m		20.6, CH_2_
5	2.85, br s; 3.46 br s	38.4, CH_2_	2.64, dd (10.0, 10.0) 3.69, br s	38.9, CH_2_	2.64, m 3.64, m		38.7, CH_2_
6-NH	5.83, br s		5.93, br s		5.78, br s		
7		171.5, C		166.6, C			166.5, C
8	2.92, dd (14.8, 9.4) 3.05, d (14.6)	41.3, CH_2_	5.83, dd (11.3, 1.3)	123.7, CH	5.82, d (11.3)		123.8, CH
9	5.49, dd (14.8, 9.4)	127.7, CH	6.08, ddd (11.3, 11.3, 2.5)	141.5, CH	6.06, dd (10.9, 10.9)		141.2, CH
10	5.34, dd (14.8, 9.4)	133.9, CH	2.39, dd (11.3, 2.5) 3.43, m	25.4, CH_2_	2.35, m 3.47, m		25.2, CH_2_
11	1.88, dd (9.4, 9.4)	56.1, CH	1.57, m	48.2, CH	1.55, dd (11.5, 11.5)		48.2, CH
12	2.30, m	47.9, CH	2.50, m	42.8, CH	2.51 m		42.5, CH
13	5.55, d (10.0)	128.4, CH	5.68, dd (10.0, 2.0)	128.0, CH	5.68, dd (10.0, 2.0)		128.6, CH
14	5.88, d (10.0)	130.9, CH	5.94, d (10.0)	131.6, CH	5.74, d (10.0)		130.0, CH
15	1.54, m	47.0, CH	1.57, m	46.9, CH	2.37, m		43.0, CH
16	1.35, m	46.9, CH	1.37, m	47.2, CH	2.68, m		58.8, CH
17	2.26, m	33.0, CH	2.26, ddd (7.4, 7.4, 7.4)	33.0, CH	2.62, m		33.7, CH
18	0.69, ddd (12.0, 12.0, 6.8) 2.11, m	38.4, CH_2_	0.69, ddd (12.0, 12.0, 6.8) 2.08, m	38.4, CH_2_	0.82, m 2.15, m		38.9, CH_2_
19	1.13, m	48.7, CH	1.16, m	48.8, CH	1.20, m		47.7, CH
20	2.10, m	42.3, CH	2.08, m	41.7, CH	2.13, m		41.0, CH
21	1.37, m 2.30, m	37.1, CH_2_	1.24, m 2.16, m	36.7, CH_2_	1.23, m 2.15, m		36.5, CH_2_
22	2.55, m	50.6, CH	2.49, m	49.5, CH	2.51, m		49.3, CH
23	6.84, dd (15.7, 9.0)	152.6, CH	6.75, dd (15.5, 10.3)	153.2, CH	6.72, dd (15.4, 10.2)	151.7, CH	
24	7.04, d (15.7)	123.2, CH	7.12, d (15.5)	122.2, CH	7.12, d (15.5)	122.4, CH	
25		174.7, C		172.9, C		172.9, C	
26		100.0, C		100.7, C		100.8, C	
27		175.5, C		173.4, C		173.4, C	
28-NR_1_			2.93, s	26.3, CH_3_	2.93,s	26.3, CH_3_	
29	0.88, d (7.2)	17.7, CH_3_	0.87, d (7.2)	17.7, CH_3_	0.85, d (7.0)	18.8, CH_3_	
30	1.35, m 1.46, m	21.6, CH_2_	1.35, m 1.46, m	21.6, CH_2_		210.2, C	
31	0.93, t (7.2)	13.2, CH_3_	0.93, t (7.2)	13.4, CH_3_	2.16, s	31.4, CH_3_	

**Figure 5:**
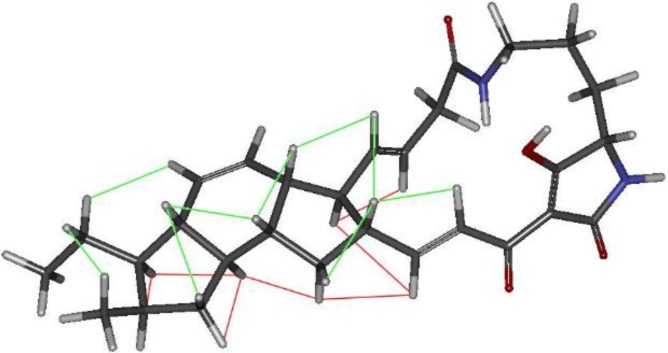
Molecular model of compound **1** showing the key observed NOESY correlations. The protons in β orientation (relative to the fused tricycle pseudoplane according to the 2D structure scketches) displaying mutual correlation are connected by green lines while red lines are employed for those in α orientation.

**Figure 6:**
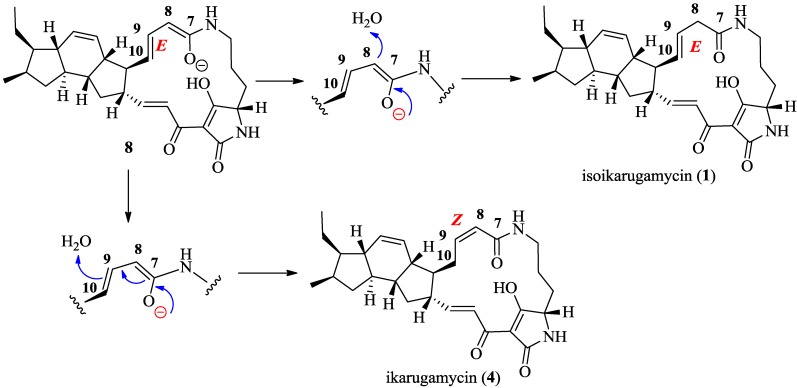
Proposed biosynthetic scheme for compound **1**.

The relative configuration of **1** was deduced to be the same as that of ikarugamycin (**4**) on the basis of the key NOESY correlations observed for compound **1** (Figure 5, Figures S5 and S6) and the common biogenetic origin of both molecules. It has been recently proposed that PTMs share a common biosynthetic pathway [18,22]. A biosynthesis route through an electrophilic addition from the corresponding intermediate **8** was proposed for ikarugamycin (**4**) [18]. Similarly, the biosynthesis of compound **1** could be explained by the deprotonation of a water molecule by the C-7/C-8 enol transient intermediate, which also justifies the *E* the stereochemistry observed for the ∆^9,10^ double bond (Figure 6). The isolation of isoikarugamycin in the current work represents the first experimental evidence validating the proposed ikarugamycin biosynthetic pathway via intermediate **8**. This is the first time that compound **1** has been isolated from a natural source and we have given it the trivial name isoikarugamycin.

Compound **2** was obtained as a white amorphous solid. Its molecular formula was assigned as C_30_H_40_N_2_O_4_ by ESI-TOFMS (*m*/*z* 493.3063, calc. for [M + H]^+^ 493.3061). The ^1^H NMR and ^13^C NMR spectra of **2** (Table 1 and Figure S2) were very similar to those of **4** (Figure S4 and Table S1, Supporting Information) indicating that it was a derivative of ikarugamycin. The singlet at δ 2.93 (3H, s) was assigned to an *N*-methyl group, being the major difference with respect to the spectrum of ikarugamycin, and indicating that compound **2** should be an *N*-methyl polycyclic tetramic acid macrolactam with ikarugamycin skeleton [18,23]. The ^13^C NMR spectrum exhibited 30 signals and the multiplicity edited HSQC spectra confirmed the presence of 5 quaternary, 15 methine, 7 methylene and 3 methyl carbons. The correlations in the HMBC experiment (Figure 4) between the *N*-methyl group at δ 2.93 and the signals at δ 66.2 (C-3), 100.7 (C-26) and 174.1 (C-27) confirmed the position of the *N*-methyl group at *N*-28 and led us to propose the structure depicted in Figure 3. The relative configuration of **2** is proposed to be identical to that reported for ikarugamycin (**4**) on the basis of NOESY correlations observed for compound **2** (see Figure S5), and the pattern of *J* values observed in its ^1^H NMR spectrum. Compound **2** has therefore the structure of 28-*N*-methylikarugamycin. This molecule has been reported previously in a patent as a synthetic derivative of ikarugamycin [24]. This is the first report on the isolation of **2** from a natural source.

The molecular formula of compound **3** was established as C_30_H_38_N_2_O_5_ by ESI-TOFMS (*m*/*z* 507.2859, calc. for [M + H]^+^ 507.2854). Its ^1^H NMR spectrum (Table 1, Figure S3) was very similar to that of compound **2**, indicating that **3** was a derivative of 28-*N*-methylikarugamycin. The most significant difference between the two spectra was the chemical shift and multiplicity of the signals due to protons of one of the methyl groups at δ 2.16 (3H, s). NMR data suggested that these two compounds differed in the presence of one carbonyl group in **3**, which must be located at C-30. The ^13^C NMR spectrum contained 30 signals: 3 methyl, 6 methylene, 15 methine and 6 quaternary carbons, according to an HSQC experiment. Four of the quaternary carbons at δ 166.5, 172.9, 173.4 and 210.2 corresponded to carbonyl groups. Correlations observed in the HMBC experiment (Figure 4) between the methyl group at δ 2.16 (H-31) and H-29 with the signal at δ 210.2 confirmed the presence of a ketone carbonyl group at C-30. On the basis of these data the structure suggested for this compound was 30-oxo-28-*N*-methylikarugamycin as shown in Figure 3. This is the first time that compound **3** has been isolated from a natural source. The *N*-demethyl derivative of **3**, clifednamide A, was recently found in cultures of an environmental *Streptomyces* sp. following a targeted polymerase chain reaction (PCR)-targeted screening approach [25].

### 2.4. Antifungal and Antibacterial Activities

The antifungal and antibacterial properties of compounds **1**–**7** were evaluated against methicillin-MRSA, *E. coli*, *C. albicans* and *A. fumigatus* (Table 2). Regarding antibacterial activity, isoikarugamycin (**1**), 28-*N*-methylikaguramycin (**2**) and ikarugamycin (**4**), showed strong inhibition of MRSA growth with MIC value ranges of 2–4, 1–2 and 2–4 μg/mL, respectively, while 30-oxo-28-*N*-methylikarugamycin (**3**) showed weaker activity against MRSA with a MIC range of 32–64 μg/mL. None of the compounds displayed activity against *E. coli* when tested at concentrations of 64 μg/mL. Concerning antifungal properties, isoikarugamycin (**1**) inhibited the growth of *C. albicans* and *A. fumigatus* with MIC value ranges of 2–4 and 4–8 μg/mL, respectively, whereas 28-*N*-methylikaguramycin (**2**) and ikarugamycin (**4**) showed a MIC value of 4 μg/mL against *C. albicans* and a MIC value range of 4–8 μg/mL against *A. fumigatus*. 30-oxo-28-*N*-methylikarugamycin **3** did not inhibit the growth of *C. albicans* or *A. fumigatus* when assayed at a concentration of 64 μg/mL. Compounds **5**–**7** did not inhibit the growth of any of the microorganisms employed in the panel when tested at a concentration of 64 μg/mL.

**Table 2:** Antibacterial and antifungal activities of compounds **1**–**7**.

Compound	MIC, μg/mL			
	MRSA MB5393	*C. albicans* MY1055	*A. fumigatus* ATCC46645	*E. coli* MB2884
isoikarugamycin (**1**)	2–4	2–4	4–8	>64
28-*N*-methylikaguramycin (**2**)	1–2	4	4–8	>64
30-oxo-28-*N*-methylikarugamycin (**3**)	32–64	>64	>64	>64
Ikarugamycin (**4**)	2–4	4	4–8	>64
MKN-003B (**5**)	>64	>64	>64	>64
1 *H*-indole-3-carboxaldehyde (**6**)	>64	>64	>64	>64
Phenylethanoic acid (**7**)	>64	>64	>64	>64

## 3. Experimental Section

### 3.1. General Experimental Procedures

Optical rotations were measured with a Jasco P-2000 polarimeter (JASCO Corporation, Tokyo, Japan). IR spectra were measured with a JASCO FT/IR-4100 spectrometer (JASCO Corporation) equipped with a PIKE MIRacle™ (JASCO Corporation) single reflection ATR accessory. NMR spectra were recorded on a Bruker Avance III spectrometer (Bruker Biospin, Fällanden, Switzerland) (500 and 125 MHz for ^1^H and ^13^C NMR, respectively) equipped with a 1.7 mm mm TCI MicroCryoProbe™ (Bruker Biospin). Chemical shifts were reported in ppm using the signals of the residual solvents as internal references (δ_H_ 7.25 and δ_C_ 77.0 for CDCl_3_). LC-UV-MS analysis was performed on an Agilent 1100 (Agilent Tehcnologies, Santa Clara, CA, USA) single quadrupole LC-MS system. ESI-TOF and MS/MS spectra were acquired using a Bruker maXis QTOF (Bruker Daltonik GmbH, Bremen, Germany) mass spectrometer coupled to an Agilent 1200 LC (Agilent Technologies, Waldbronn, Germany). Acetone used for extraction was analytical grade. Solvents employed for isolation were HPLC grade. Molecular models were generated in Chem3D Pro 12.0 (CambridgeSoft, PerkinElmer Informatics, Waltham, MA, USA). Torsional angles of the C11-C10 and C22-C23 bonds outside the fused tricycle were first adjusted manually to reasonable values which could explain the key NOEs observed for protons H-9, H-10, H-23 and H-24. Those starting structures were minimized by molecular mechanics with the MM2 force field using as gradient convergence criteria an RMS value of 0.001. The resulting models were in agreement with that reported for butremycin [23] and perfectly accounted for all the key NOEs observed in the spectra.

### 3.2. Fermentation of the Producing Microorganism

A 1 liter fermentation of strain CA-185989 was generated as follows: a seed culture of the strain was obtained by inoculating three 50 mL tubes each containing 14 mL of ATCC-2-M medium (soluble starch 20 g/L, glucose 10 g/L, NZ Amine Type E 5 g/L, meat extract 3 g/L, peptone 5 g/L, yeast extract 5 g/L, sea salts 30 g/L, calcium carbonate 1 g/L, pH 7) with 0.7 mL of a freshly thawed inoculum stock of the producing strain. Tubes were incubated in a rotary shaker at 28 °C, 70% relative humidity and 220 rpm for about 96 h. This fresh inoculum was employed to inoculate twenty 250 mL flasks, each containing 50 mL of APM9-modifed medium (2.5% v/v) (glucose 50 g/L, soluble starch 12 g/L, soy flour 30 g/L, CoCl_2_·6H_2_O 2 mg/L, sea salts 30 g/L, calcium carbonate 7 g/L, pH 7). The inoculated flasks were incubated in a rotary shaker at 28 °C, 70% relative humidity and 220 rpm for 6 days before harvesting.

### 3.3. Extraction and Bioassay Guided Isolation

The scaled-up fermentation broth (1 L) was extracted with acetone (1 L) under continuous shaking at 220 rpm for 2 h. The mycelium was separated by filtration and the supernatant (*ca.* 2 L) was concentrated to 1 L under reduced pressure. This aqueous residue was extracted with ethyl acetate (A). 10 mL of the resulting aqueous layer were dried under reduced pressure (B). Bioactivity against *A. fumigatus*, *C. albicans* and MRSA was confirmed in the ethyl acetate extract. This extract (A, 3.94 g) was chromatographed on SiO_2_ using mixtures of hexane/EtOAc of increasing polarity and methanol to afford ten fractions: A1-A10. Fractions A4 (0.020 g), A8 (0.75 g) and A10 (1.05 g) were the most active against *A. fumigatus* and *C. albicans*.

Fraction A4 (0.020 g, hexane/EtOAc, 1:1) was subjected to fractionation by reversed-phase semipreparative HPLC (column Agilent Zorbax RX-C8, 9.4 × 250 mm, 7 μm; 3 mL·min^−1^, UV detection at 210 and 280 nm) with a linear gradient of CH_3_CN/H_2_O with 0.1% trifluoroacetic acid from 10% to 40% CH_3_CN over 36 min yielding **6** (1.2 mg, *t*_R_ 28 min) and **7** (1.8 mg, *t*_R_ 32 min).

Fraction A8 (0.750 g, hexane/EtOAc, 1:4–1:9) was selected for further fractionation by reversed-phase preparative HPLC (column Agilent Zorbax SB-C8, 21.2 × 250 mm, 7 μm; 20 mL·min^−1^, UV detection at 210 and 280 nm) with a linear gradient of CH_3_CN/ H_2_O with 0.1% trifluoroacetic acid, from 20% to 85% CH_3_CN over 45 min, to afford seven subfractions: A8A-A8G. Subfractions A8D, A8E, A8F and A8G were the most active against *A. fumigatus* and *C. albicans*. Subfraction A8D was chromatographed on Sephadex LH-20 using mixtures of chloroform/methanol (2:1) affording four fractions. The third fraction was purified by reverse-phase semipreparative HPLC (column Agilent Zorbax RX-C8, 9.4 × 250 mm, 7 μm; 3 mL·min^−1^, UV detection at 210 and 280 nm) with an isocratic solvent system of CH_3_CN/H_2_O (45/55) with 0.1% trifluoroacetic acid to yield **3** (3.2 mg, *t*_R_ 27 min). Subfractions A8E and A8G were further purified by reverse-phase semipreparative HPLC (column Agilent Zorbax RX-C8, 9.4 × 250 mm, 7 μm; 3 mL·min^−1^, UV detection at 210 and 280 nm) with linear gradient of CH_3_CN/ H_2_O with 0.1% trifluoroacetic acid, from 50% to 80% CH_3_CN yielding **4** (2.2 mg, *t*_R_ 18 min) and **2** (2.2 mg, *t*_R_ 25 min), respectively. Subfraction A8F was subjected to CC on Sephadex LH-20 using a mixture of CHCl_3_/MeOH (2:1). Further purification by C-8 HPLC under the same chromatographic conditions, yielded **4** (3.2 mg, *t*_R_ 25 min) and **1** (0.8 mg, *t*_R_ 26 min).

Fraction A10 (0.120 g, MeOH) was subjected to fractionation by reversed-phase preparative HPLC (column Agilent Zorbax SB-C8, 21.2 × 250 mm, 7 μm; 20 mL·min^−1^, UV detection at 210 and 280 nm) with linear gradient CH_3_CN/H_2_O with 0.1% trifluoroacetic acid from 30% to 60% CH_3_CN, yielding **5** (2.5 mg, *t*_R_ 11 min) and **4** (3.5 mg, *t*_R_ 35 min).

Isoikarugamycin (**1**): White amorphous solid; [α]^25^_D_ +362.1 (c 0.085, CHCl_3_); UV (DAD) λ_max_ 228, 323 nm; IR (ATR) ν_max_ 3319, 2955, 1715, 1647, 962 cm^−1^; ^1^H and ^13^C NMR: See Table 1; (+)-HRESIMS *m*/*z* 479.2899 [M + H]^+^ (calc. for C_29_H_39_N_2_O_4_^+^, 479.2904).

28-*N*-methylikarugamycin (**2**): White amorphous solid; [α]^25^_D_ + 141.3 (c 0.01, CHCl_3_); UV (DAD) λ_max_ 228, 330 nm; IR (ATR) ν_max_ 3339, 2953, 1715, 1639, 939 cm^−1^; ^1^H and ^13^C NMR: see Table 1; (+)-HRESIMS *m*/*z* 493.3063 [M + H]^+^ (calc. for C_30_H_41_N_2_O_4_^+^, 493.3061).

30-oxo-28-N-methylikarugamycin (**3**): Pale pink amorphous solid; [α]^25^_D_ +275.5 (c 0.095, CHCl_3_); UV (DAD) λ_max_ 228, 335 nm; IR (ATR) ν_max_ 3341, 2918, 1704, 1636, 989 cm^−1^; ^1^H and ^13^C NMR: See Table 1; (+)-HRESIMS *m*/*z* 507.2859 [M + H]^+^ (calc. for C_30_H_39_N_2_O_5_^+^, 507.2854).

### 3.4. Antifungal and Antibacterial Assays

Compounds **1**–**7** were tested for their ability to inhibit the growth of key fungi (*A. fumigatus* ATCC46645), yeast (*C. albicans* MY1055) and bacteria (*E. coli* MB2884 and meticillin-resistant *S. aureus* MB5393) following a previously described methodology [26,27]. Briefly, each compound was serially diluted in DMSO with a dilution factor of 2 to provide 10 concentrations starting at 64 μg/mL for all the assays. The MIC was defined as the lowest concentration of an antibacterial or antifungal compound that inhibited ≥95% of the growth of a microorganism after overnight incubation. The Genedata Screener software (version 11.0.3, Genedata, Inc., Basel, Switzerland) was used to process and analyze the data and also to calculate the RZ’ factor which predicts the robustness of an assay [28]. In all experiments performed in this work the RZ’ factor obtained was between 0.87 and 0.98.

## 4. Conclusions

Ikarugamycin and three new bioactive polycyclic tetramate macrolactams **1**–**3** have been identified from the culture of an actinomycete strain closely related to * Streptomyces zhaozhouensis* NEAU-LZS-5(T), isolated from a marine sediment sample collected in Equatorial Guinea. Compounds **1**–**2** caused strong inhibition of the growth of methicillin-resistant *S. aureus*, *C. albicans* and *A. fumigatus*, with MIC values in the micromolar range. MIC values obtained for the series of PTMs suggest that whereas *N*-methylation of the nitrogen atom of the tetramic acid moiety does not affect the antibacterial and antifungal activity of the compounds, the presence of a carbonyl group in the ethyl side chain at C-16 causes a severe decrease in the MIC revealing that the presence of the ethyl group plays a key role in the biological activity of this family of molecules. This article constitutes the first report on the chemical composition of extracts from a marine strain closely related to the recently described terrestrial species *S. zhaozhouensis* [4] and confirms that marine derived actinomycetes continue to be a rich and underexploited source of new small molecules that could lead to the discovery of new antibiotics.

## Supplementary Files

Supplementary File 1
