# Supplementary material for: New Ikarugamycin Derivatives with Antifungal and Antibacterial Properties from Streptomyces zhaozhouensis
Source: Mar Drugs. 2014 Dec 29;13(1):128–40. doi: 10.3390/md13010128 (PMC4306928; doi:10.3390/md13010128)
Supplement: Supplementary File 1 [file marinedrugs-13-00128-s001.pdf]

Supplementary Information

Figure S1. Spectroscopic data for compound **1**. (**A**) ESI-TOF and UV spectra for compound **1**; (**B**) ^{1}H NMR spectrum ($CDCl_3$, 500 MHz) of compound **1**; (**C**) ^{13}C NMR spectrum ($CDCl_3$, 125 MHz) of compound **1**; (**D**) COSY spectrum of compound **1**; (**E**) HSQC spectrum of compound **1**; (**F**) HMBC spectrum of compound **1**; **(G**) NOESY spectrum of compound **1**.

Figure S2. Spectroscopic data for compound **2**. (**A**) ESI-TOF and UV spectra for compound **2**; (**B**) ^{1}H NMR spectrum ($CDCl_3$, 500 MHz) of compound **2**; (**C**) ^{13}C NMR spectrum ($CDCl_3$, 125 MHz) of compound **2**; (**D**) COSY spectrum of compound **2**; (**E**) HSQC spectrum of compound **2**; (**F**) HMBC spectrum of compound **2**; **(G**) NOESY spectrum of compound **2**.

Figure S3. Spectroscopic data for compound **3**. (**A**) ESI-TOF and UV spectra for compound **3**; (**B**) ^{1}H NMR spectrum ($CDCl_3$, 500 MHz) of compound **3**; (**C**) ^{13}C NMR spectrum ($CDCl_3$, 125 MHz) of compound **3**; (**D**) COSY spectrum of compound **3**; (**E**) HSQC spectrum of compound **3**; (**F**) HMBC spectrum of compound **3**; **(G**) NOESY spectrum of compound **3**.

Figure S4. NMR spectra of ikaguramycin (**4**). (**A**) ^{1}H NMR spectrum ($CDCl_3$, 500 MHz) of compound **4**; (**B**) ^{13}C NMR spectrum ($CDCl_3$, 125 MHz) of compound **4**; (**C**) NOESY spectrum of compound **4**.

Figure S5. Molecular models of **1**–**4** showing the key observed NOEs.

Figure S6. Overlay of the molecular models of **1**–**4**.

Table S1. NMR data of ikarugamycin (**4**).

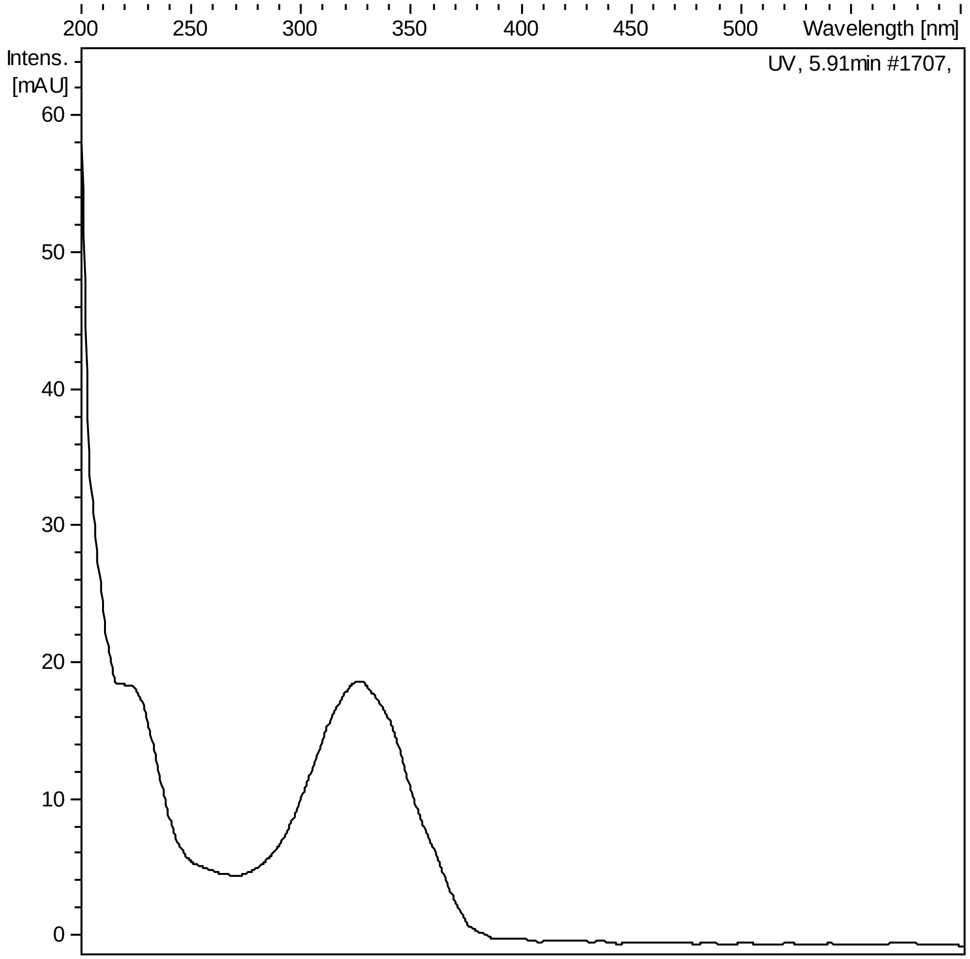

UV spectrum of compound **1**.

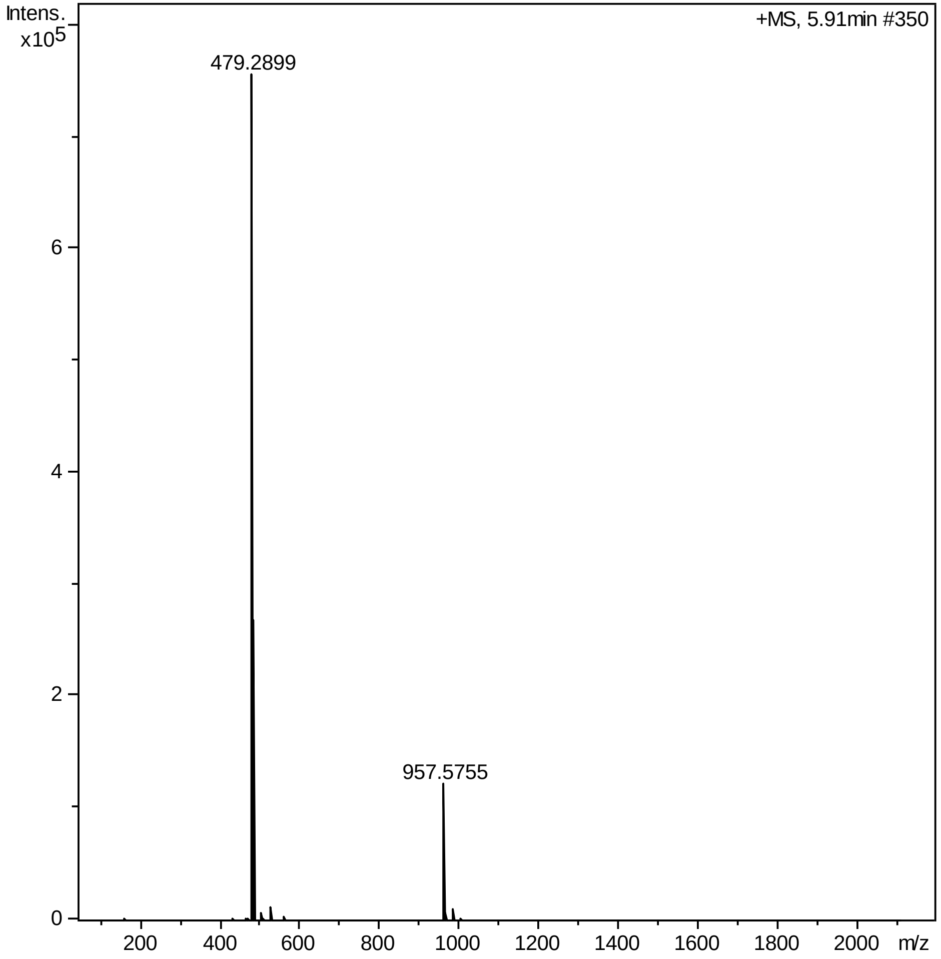

ESI-TOF spectrum of compound **1**.

(**A**)

Figure S1. *Cont.*

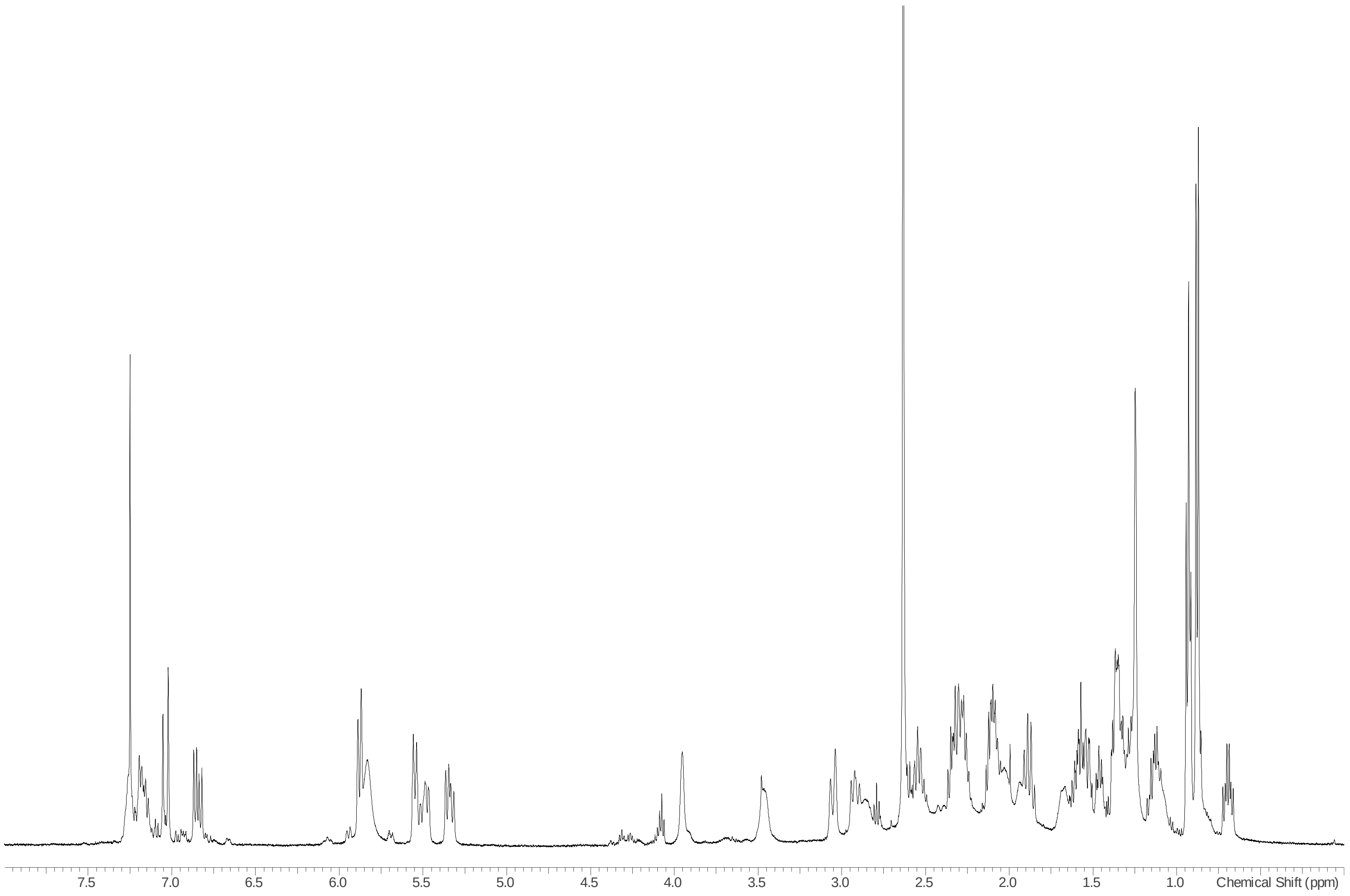

Figure S1. *Cont.*

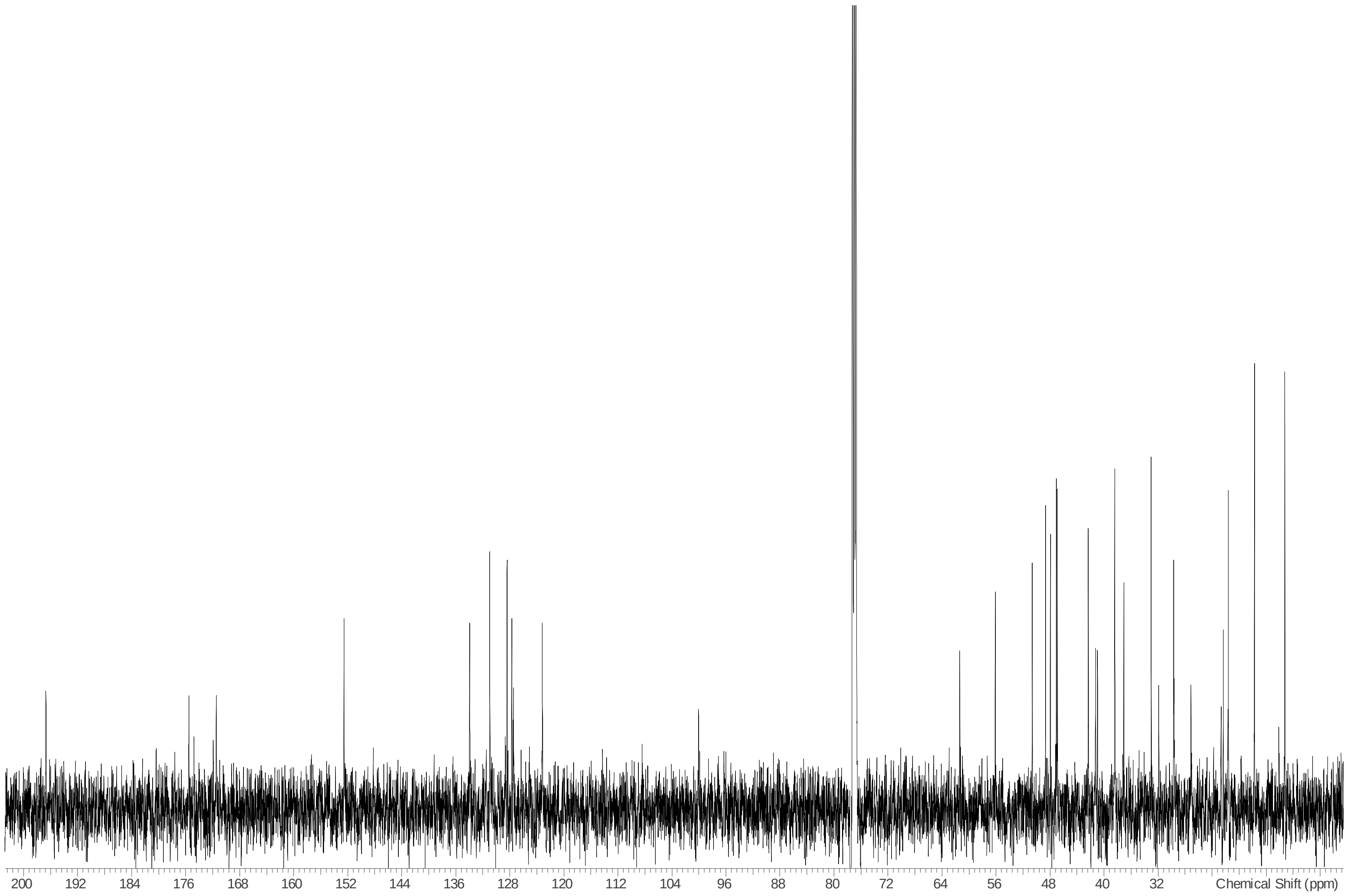

Figure S1. *Cont.*

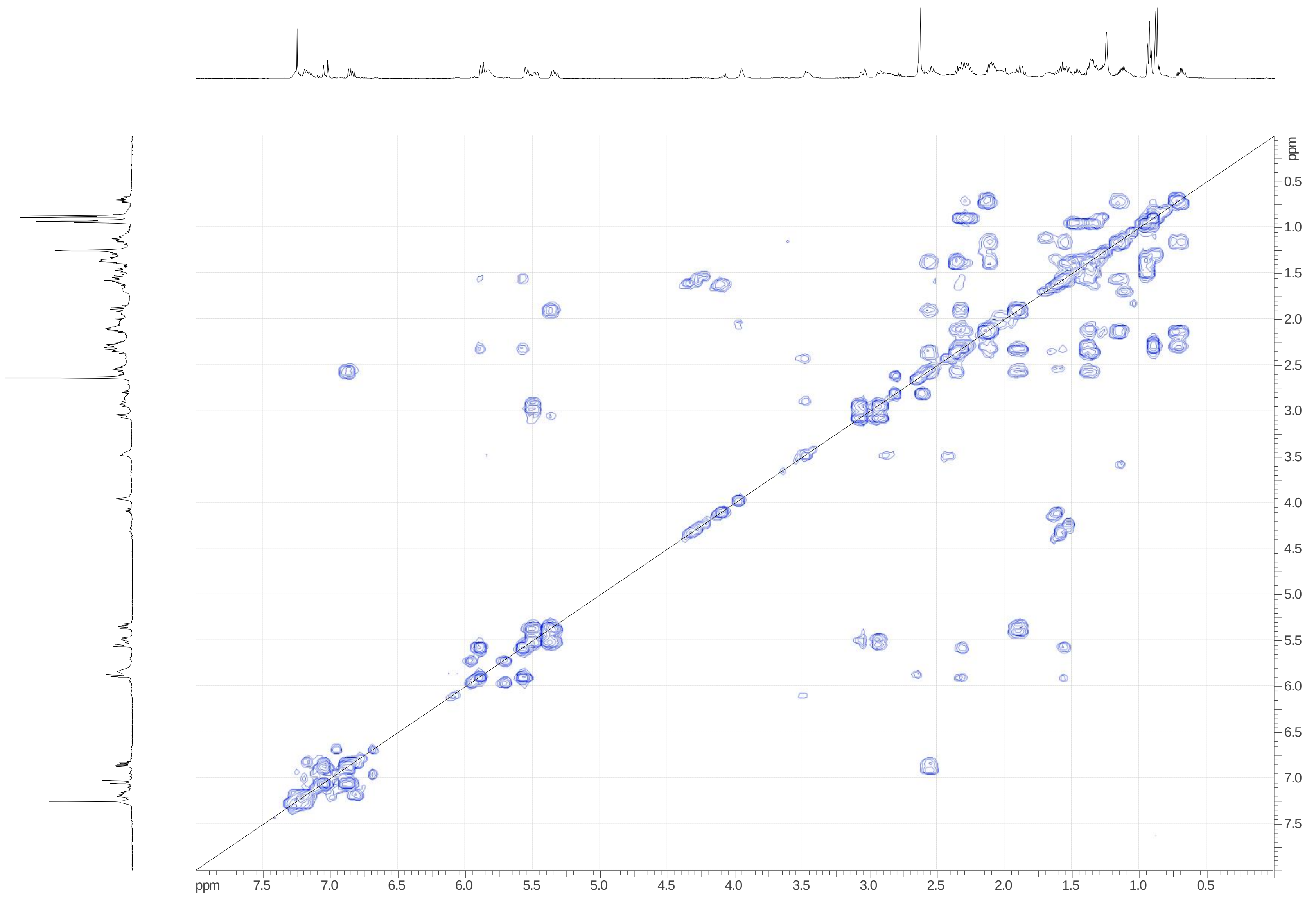

Figure S1. *Cont.*

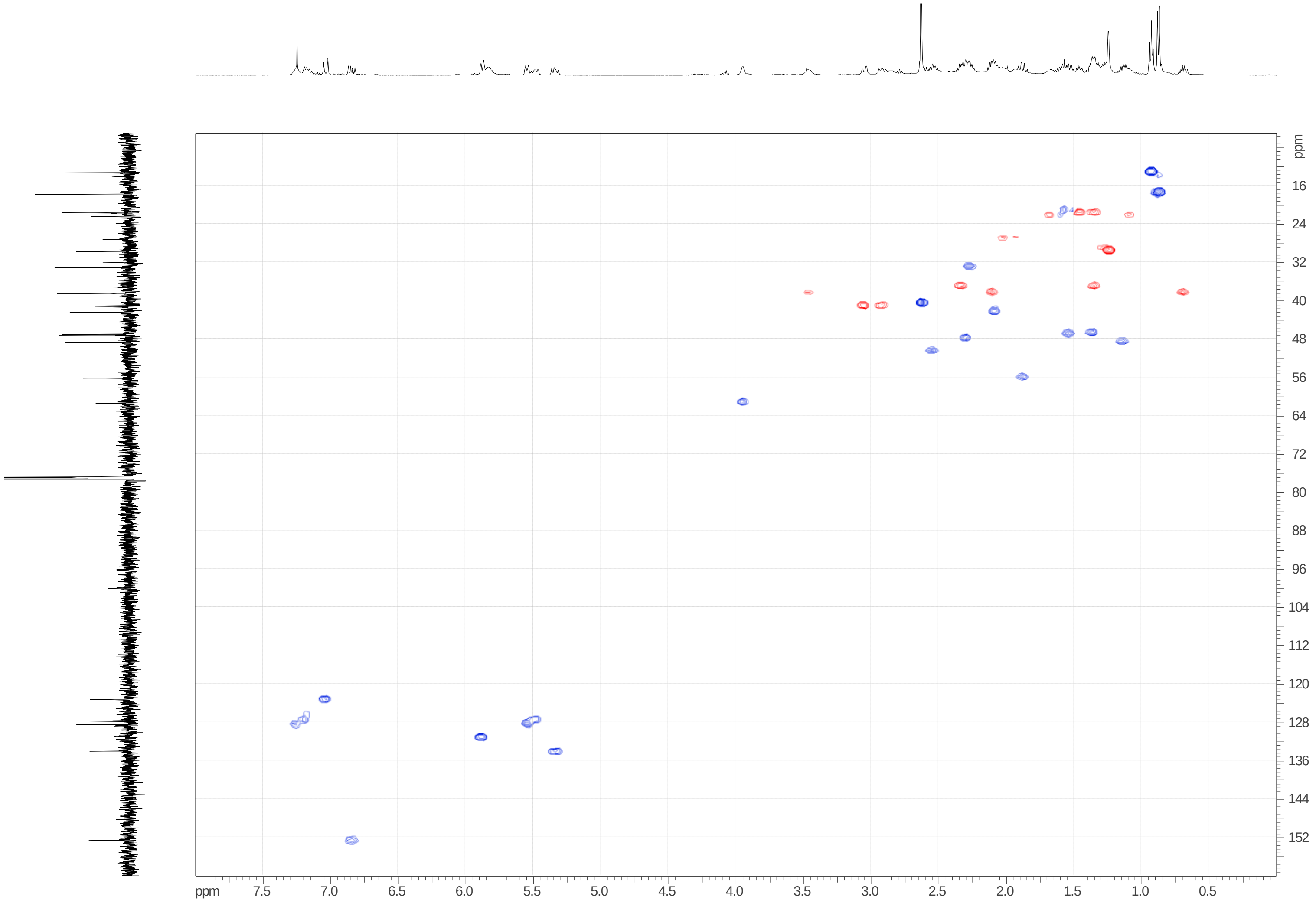

Figure S1. *Cont.*

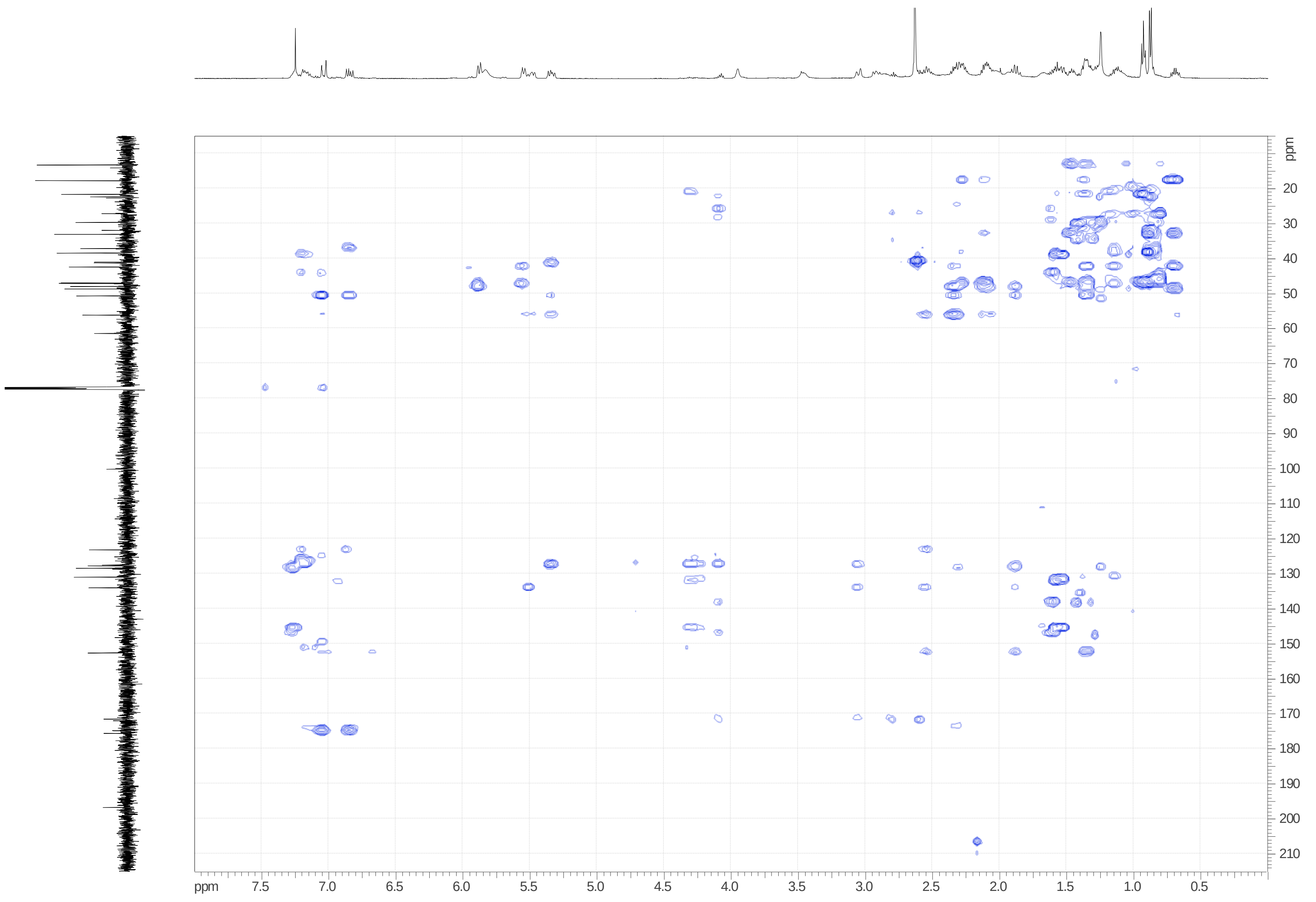

Figure S1. *Cont.*

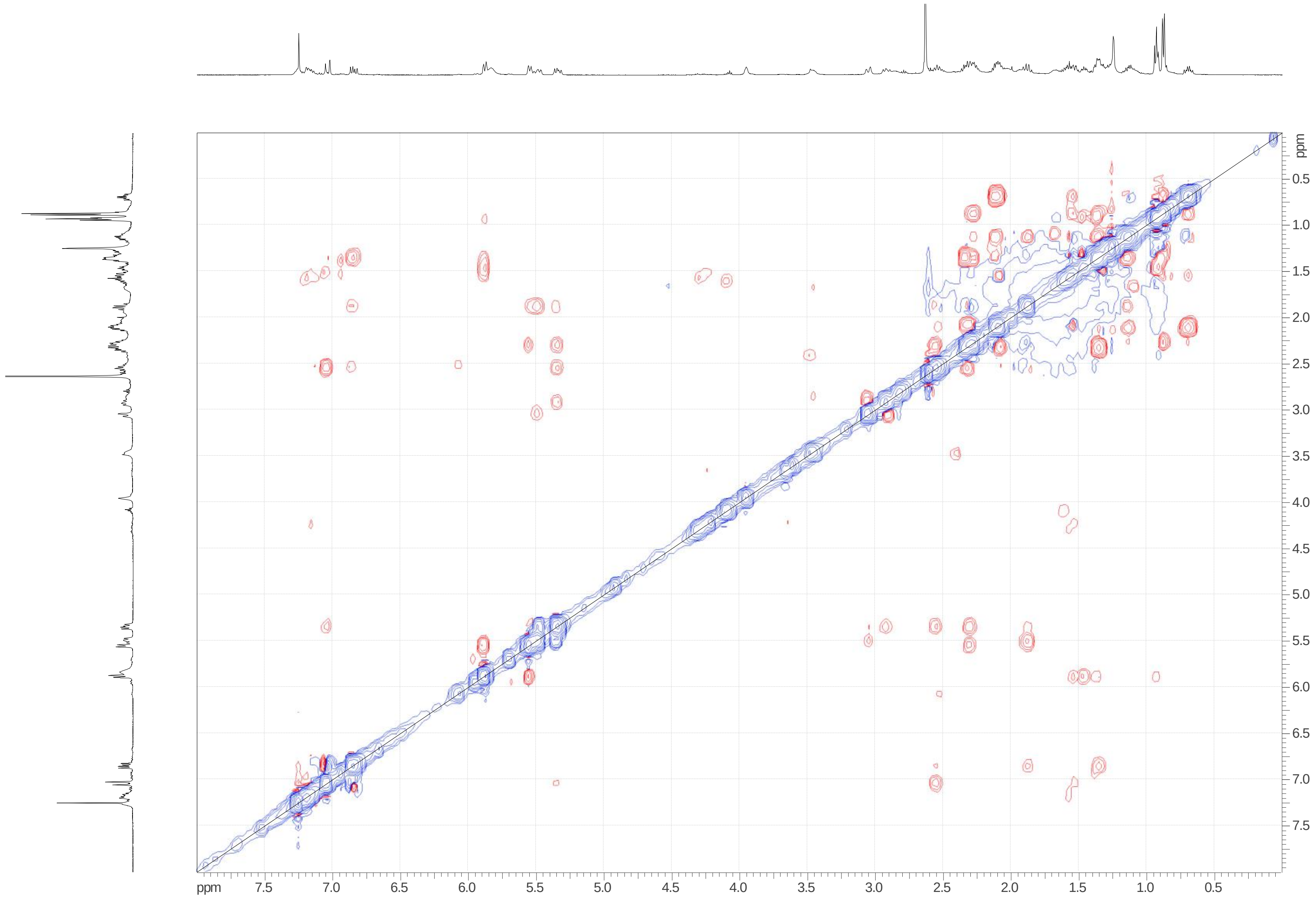

Figure S1. (**A**) ESI-TOF and UV spectra of compound **1**; (**B**) ^{1}H NMR ($CDCl_3$, 500 MHz) of compound **1**; (**C**) ^{13}C NMR ($CDCl_3$, 125 MHz) of compound **1**; (**D**) COSY of compound **1**; (**E**) HSQC of compound **1**; (**F**) HMBC of compound **1**. (**G**) NOESY of compound **1**.

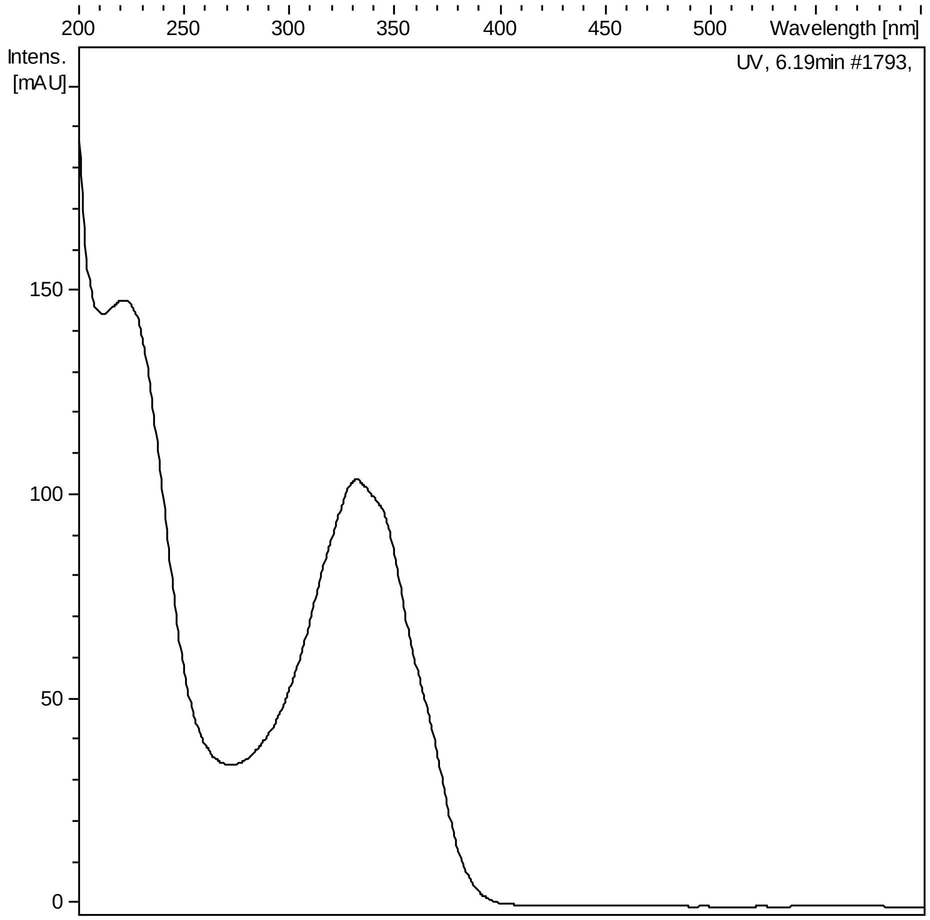

UV spectrum of compound **2**.

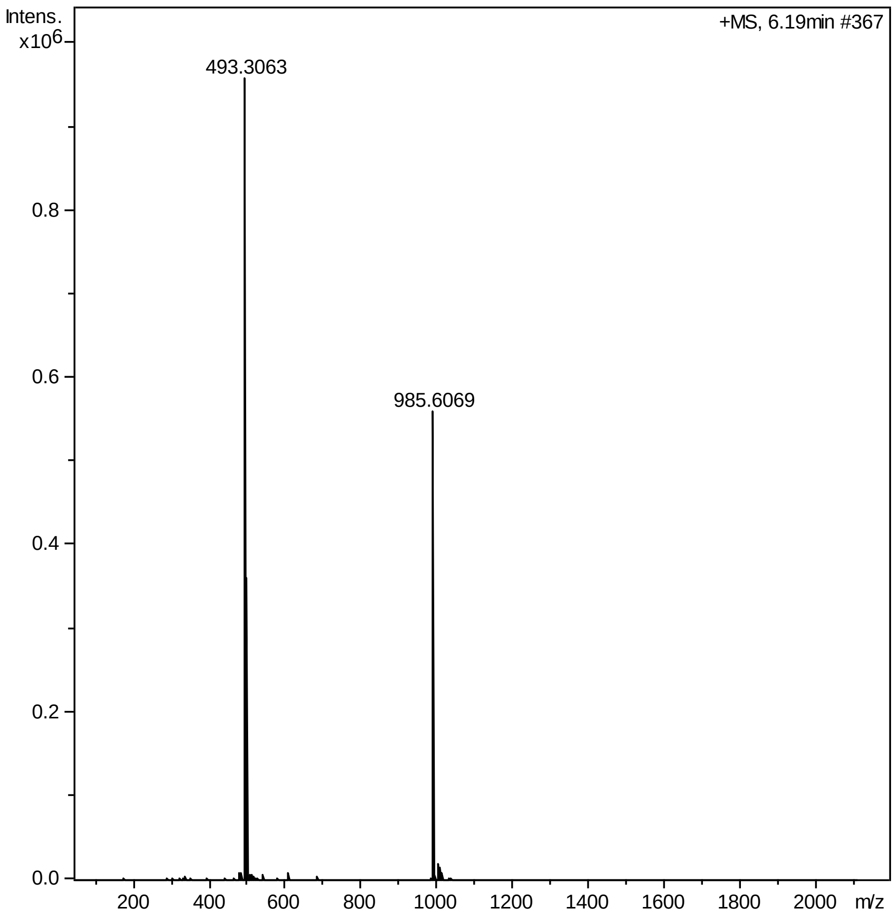

ESI-TOF spectrum of compound **2**.

Figure S2. *Cont.*

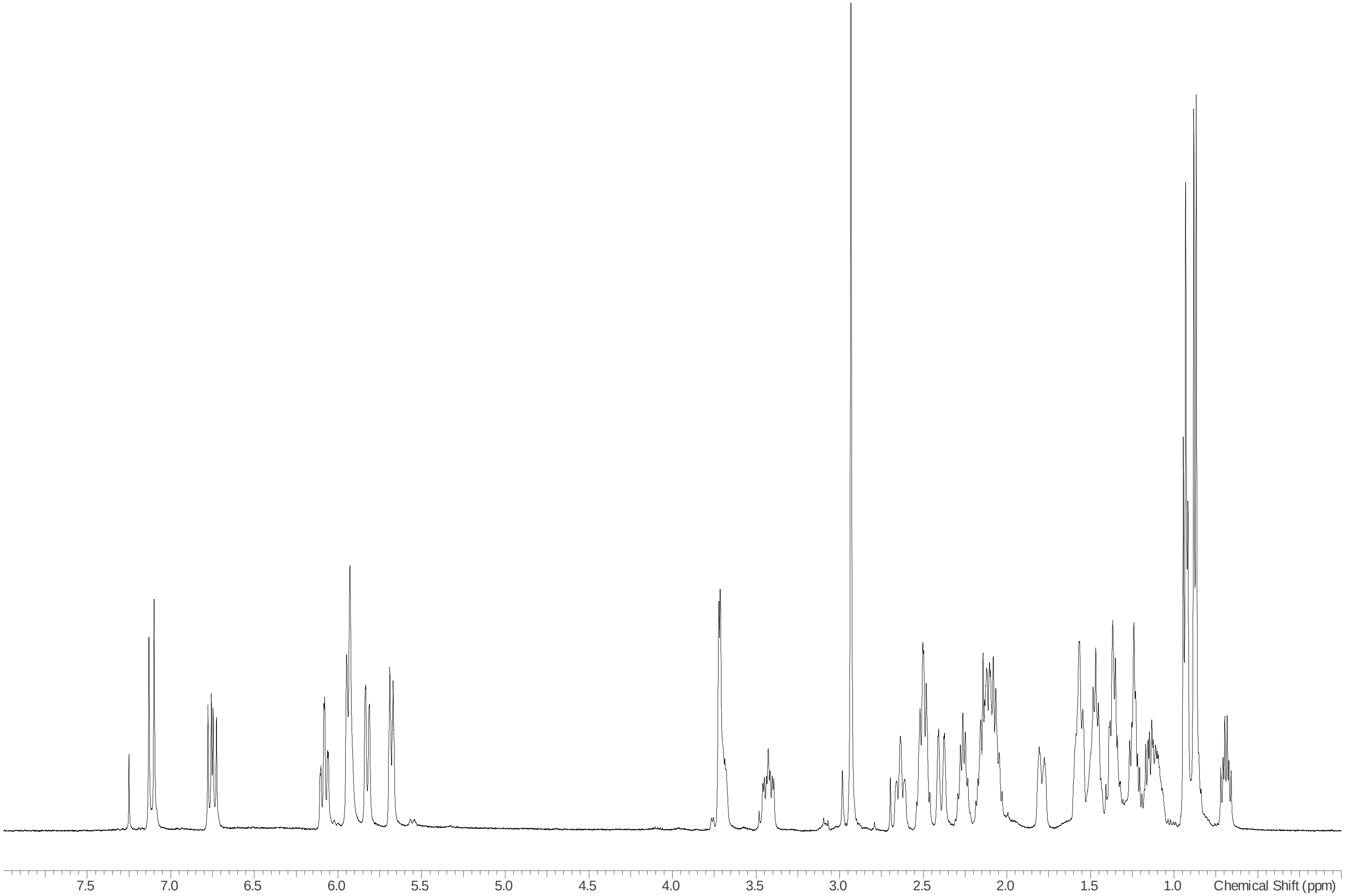

Figure S2. *Cont.*

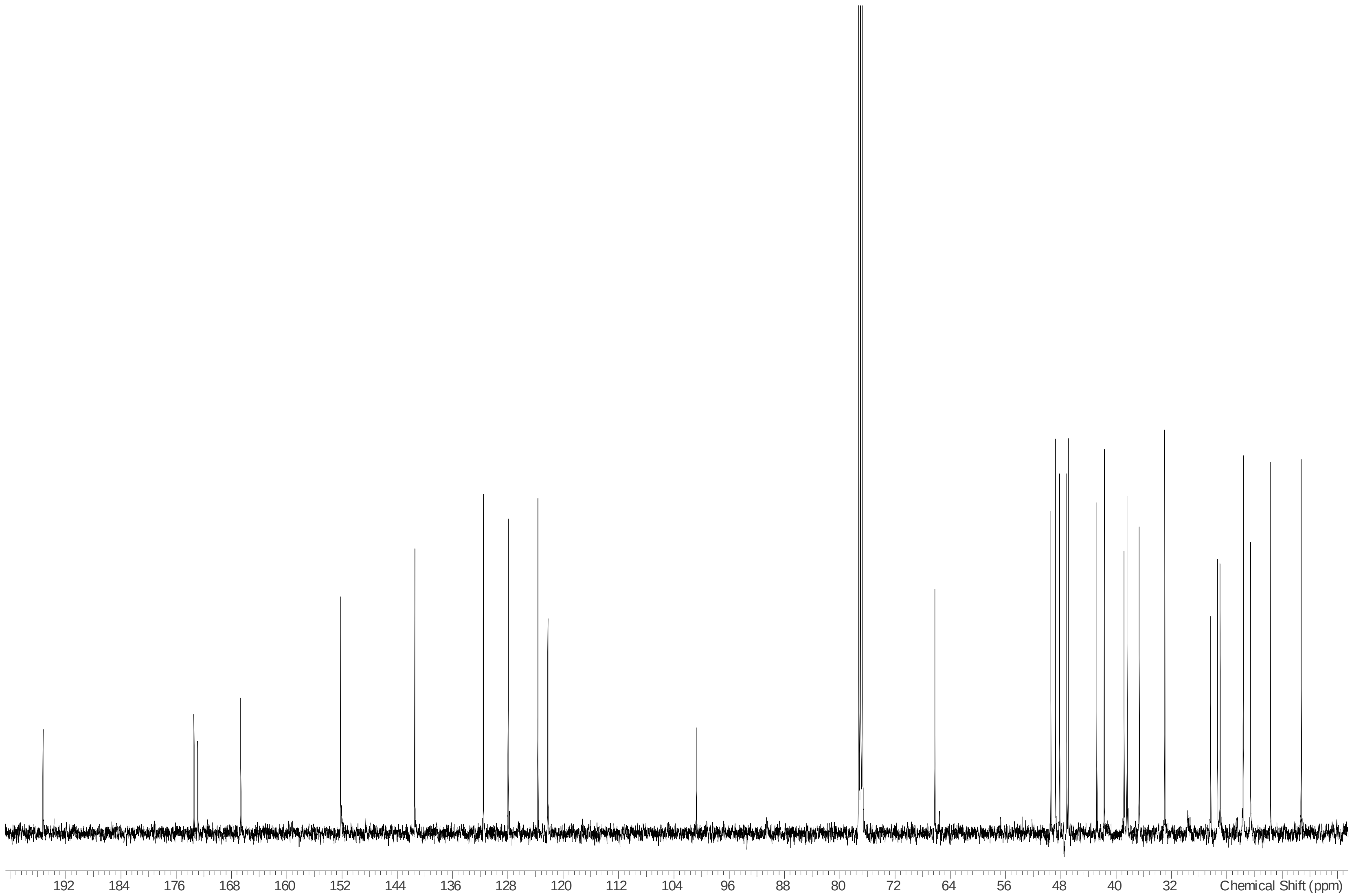

Figure S2. *Cont.*

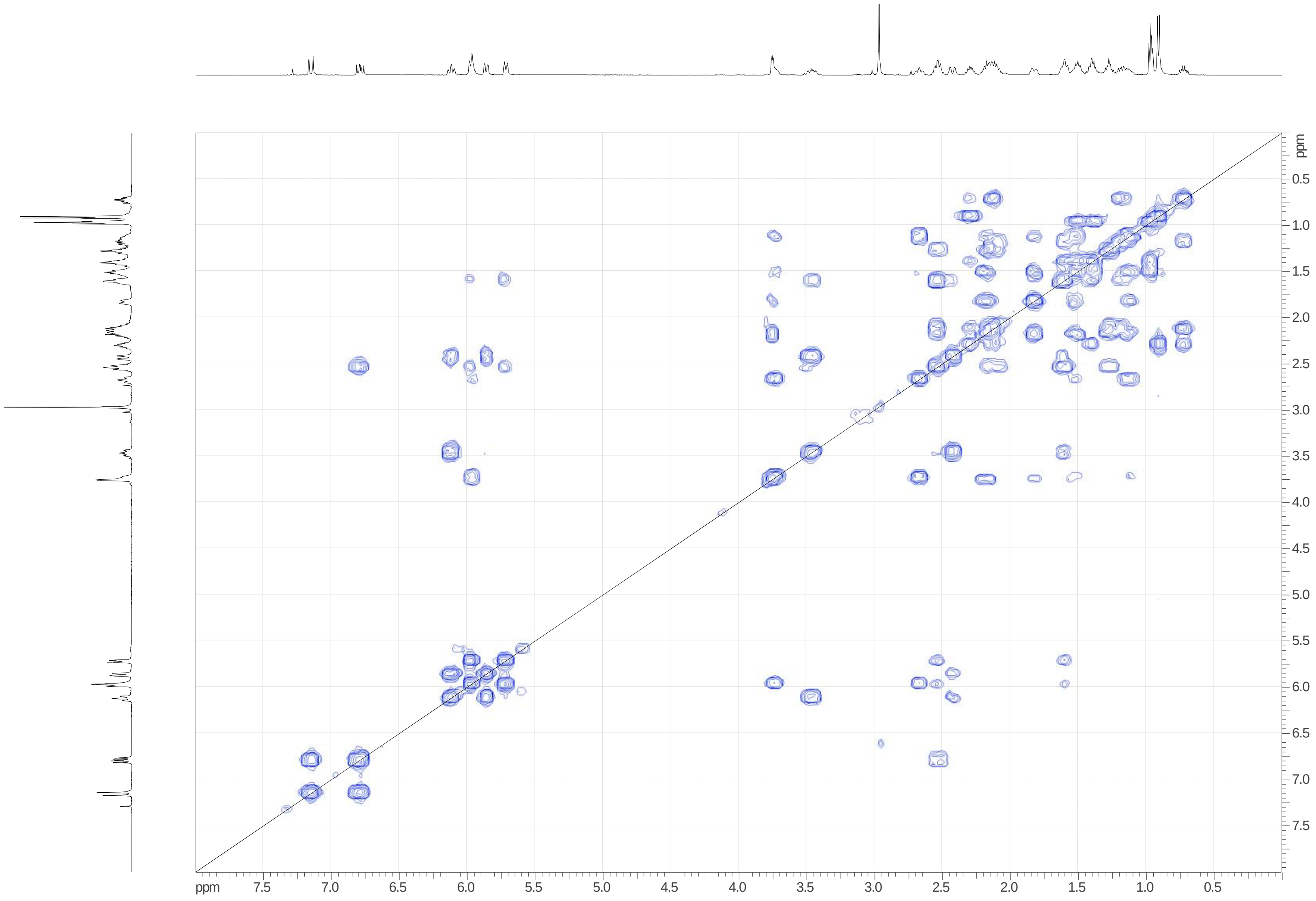

Figure S2. *Cont.*

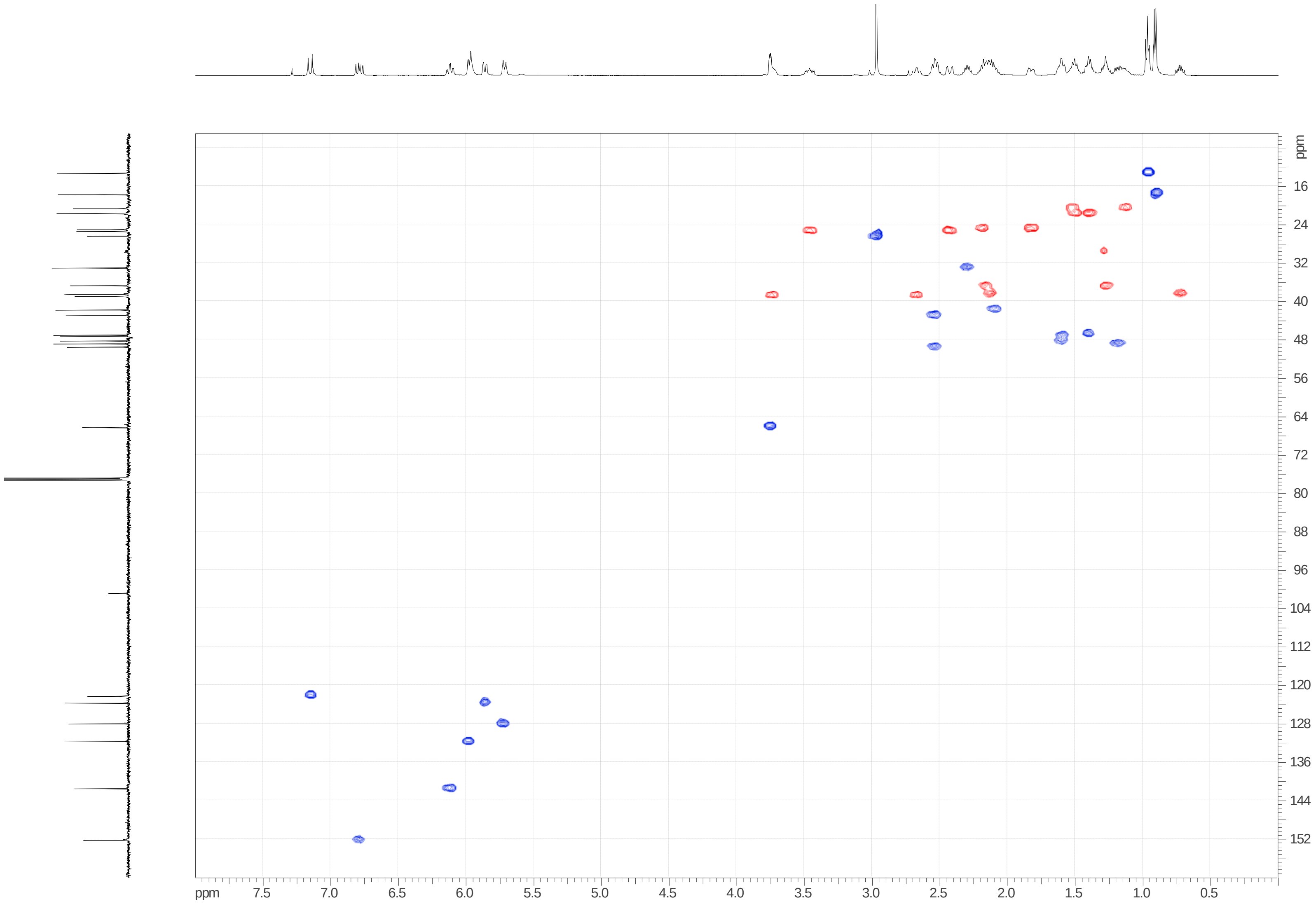

Figure S2. *Cont.*

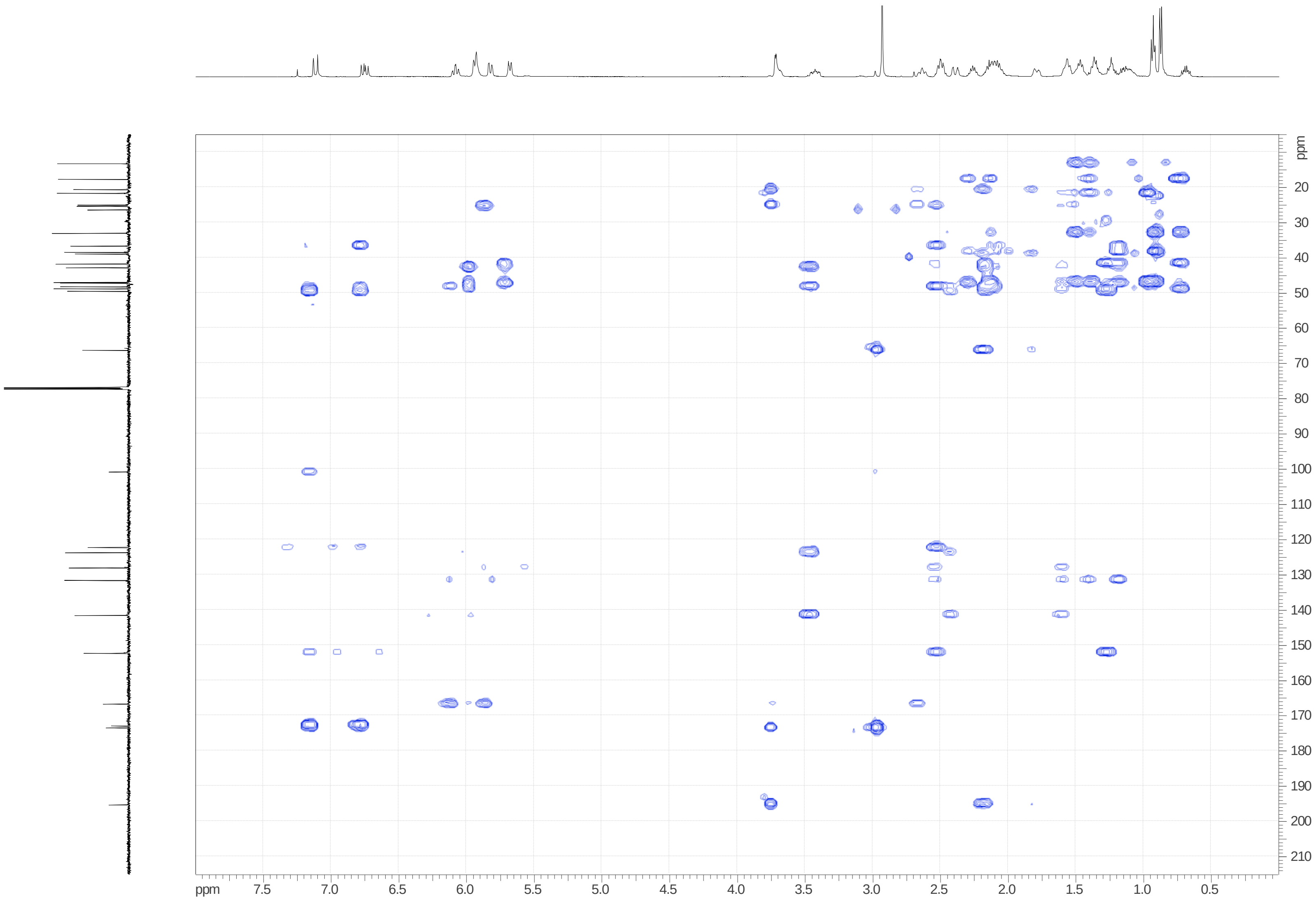

Figure S2. *Cont.*

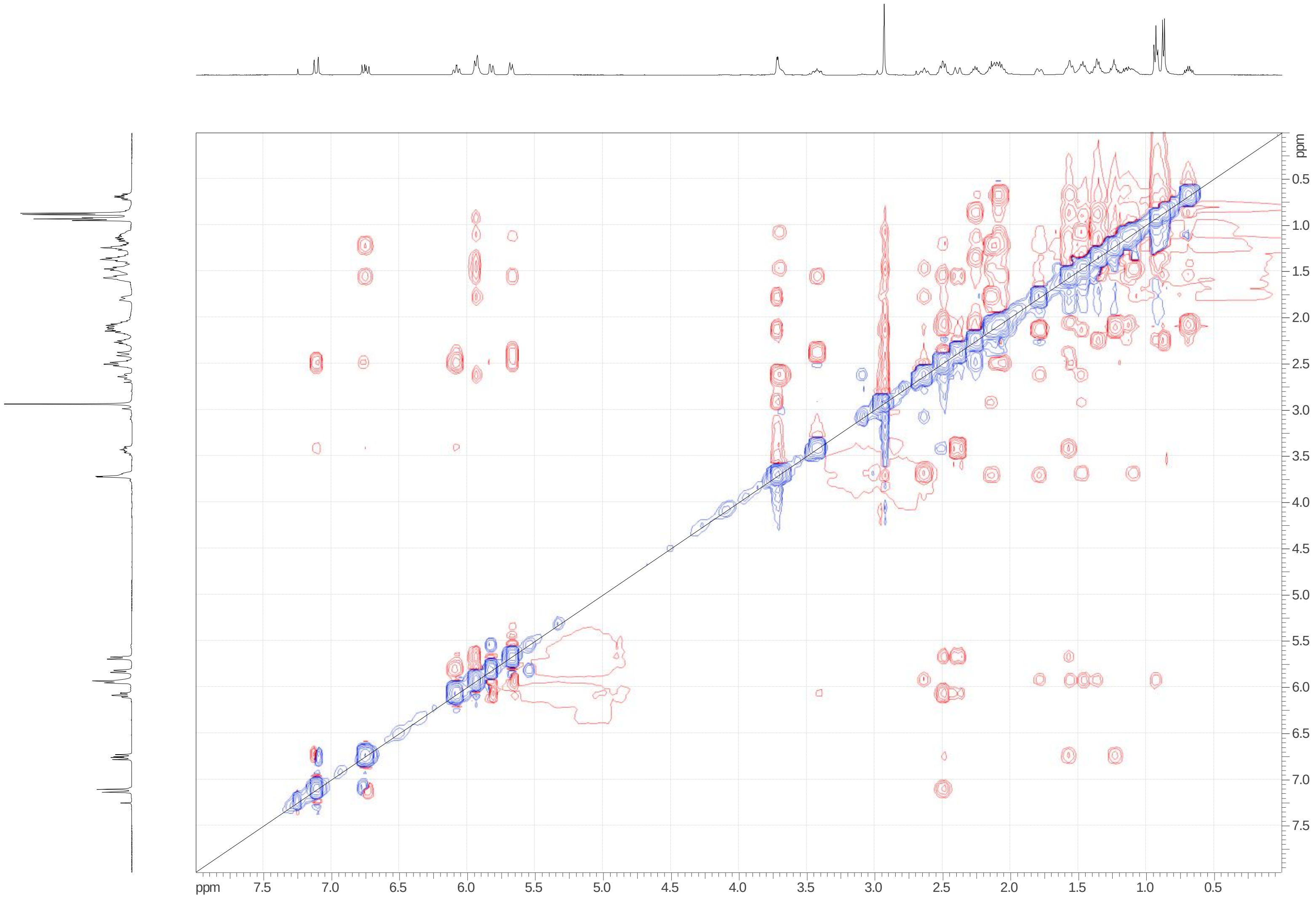

Figure S2. (**A**) ESI-TOF and UV spectra of compound **2**; (**B**) ^{1}H NMR ($CDCl_3$, 500 MHz) of compound **2**; (**C**) ^{13}C NMR ($CDCl_3$, 125 MHz) of compound **2**; (**D**) COSY of compound **2**. (**E**) HSQC of compound **2**; (**F**) HMBC of compound **2**. (**G**) NOESY of compound **2**.

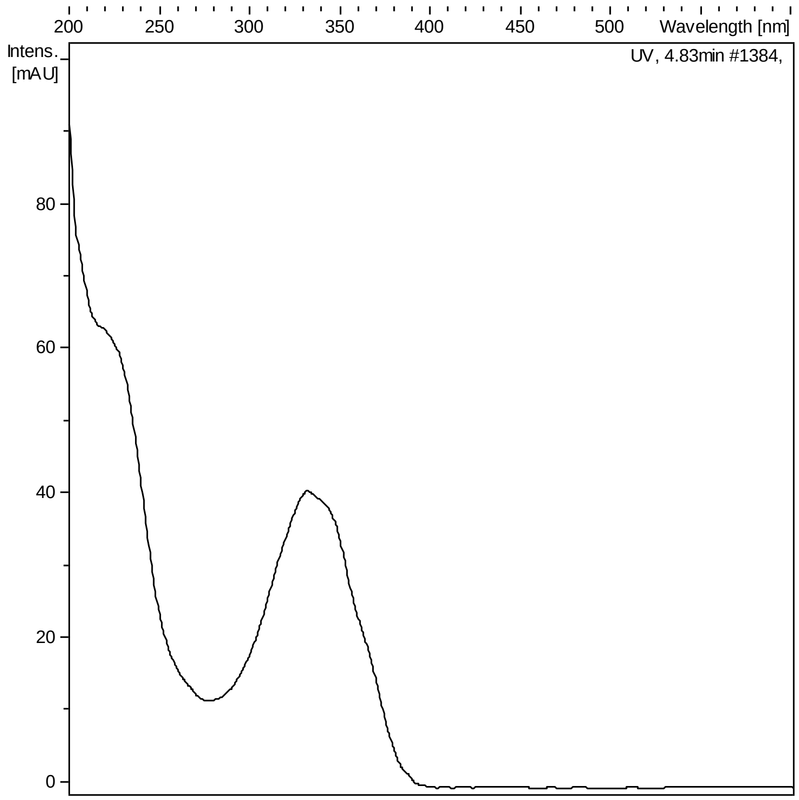

UV spectrum of compound **3**.

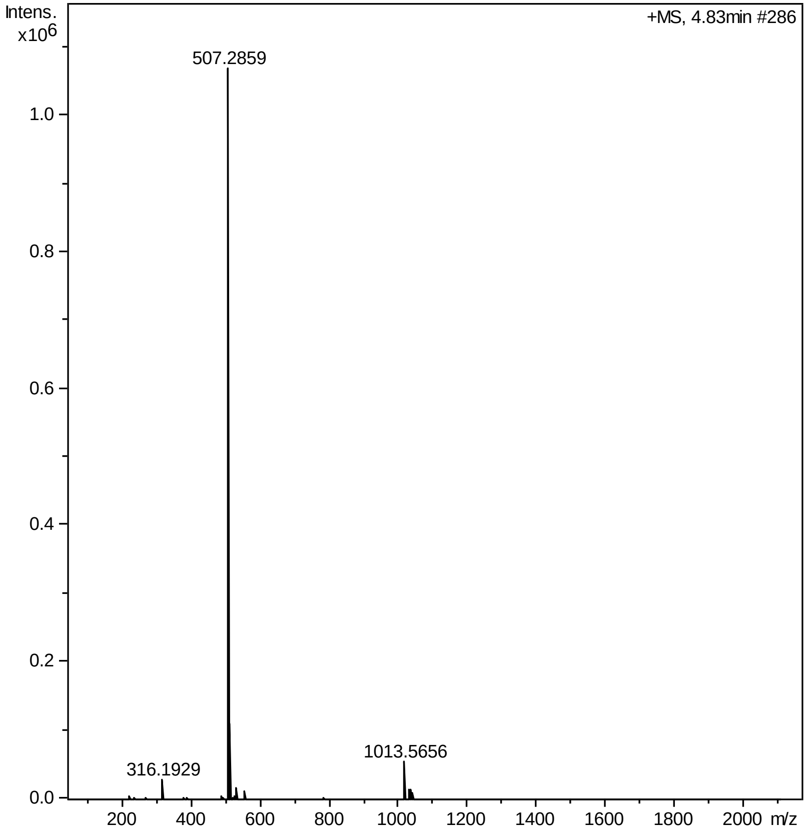

ESI-TOF spectrum of compound **3**.

Figure S3. *Cont.*

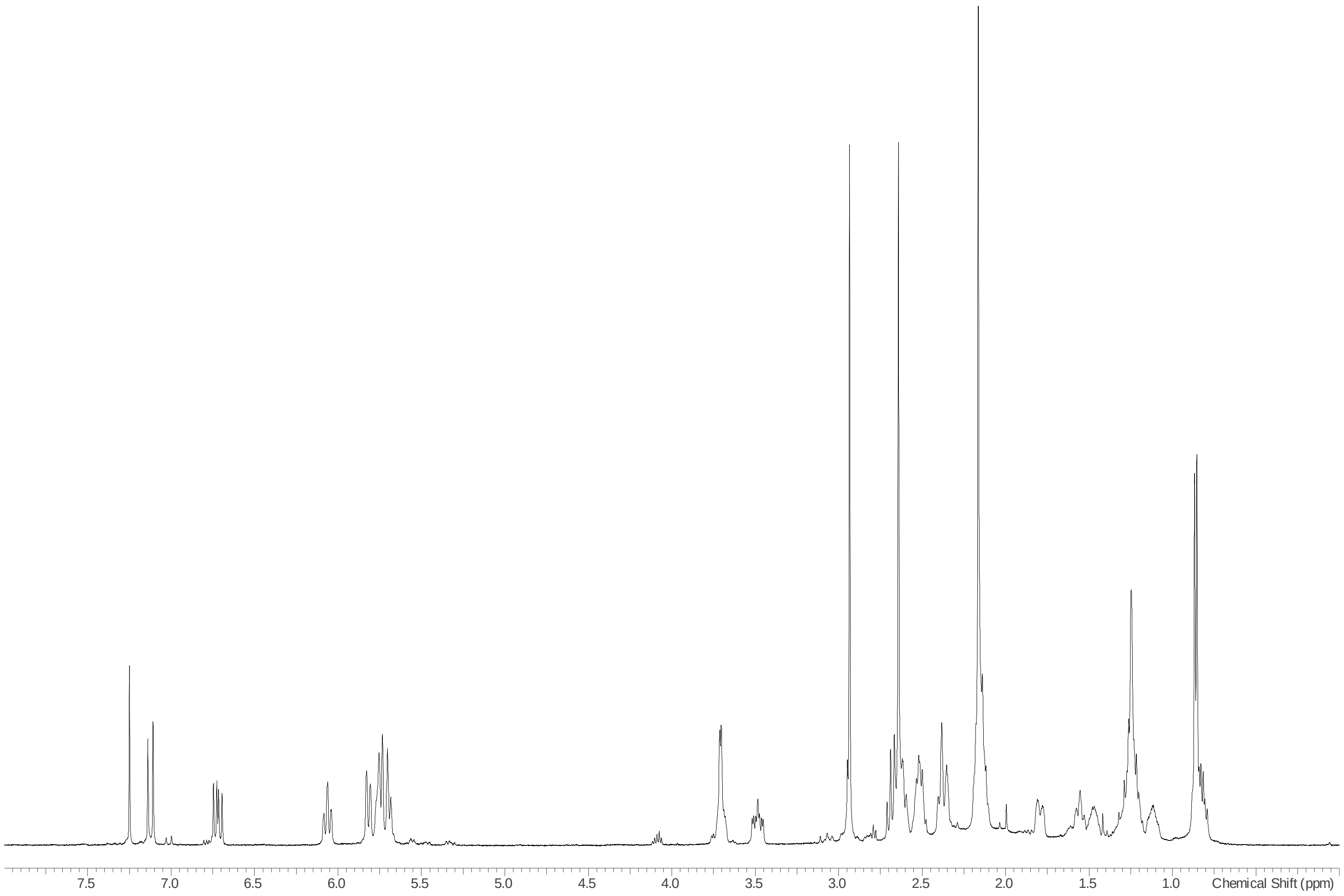

Figure S3. *Cont.*

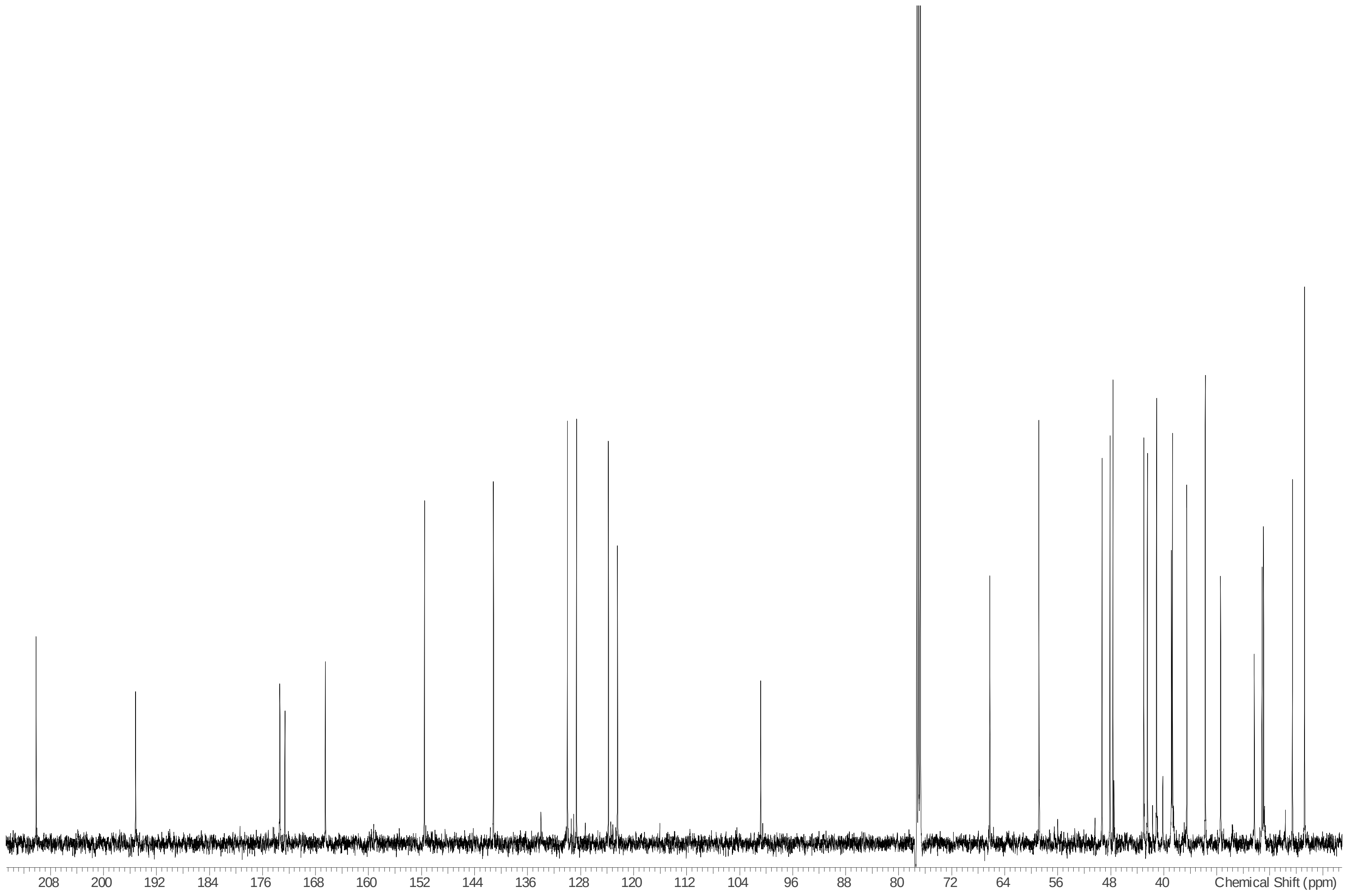

Figure S3. *Cont.*

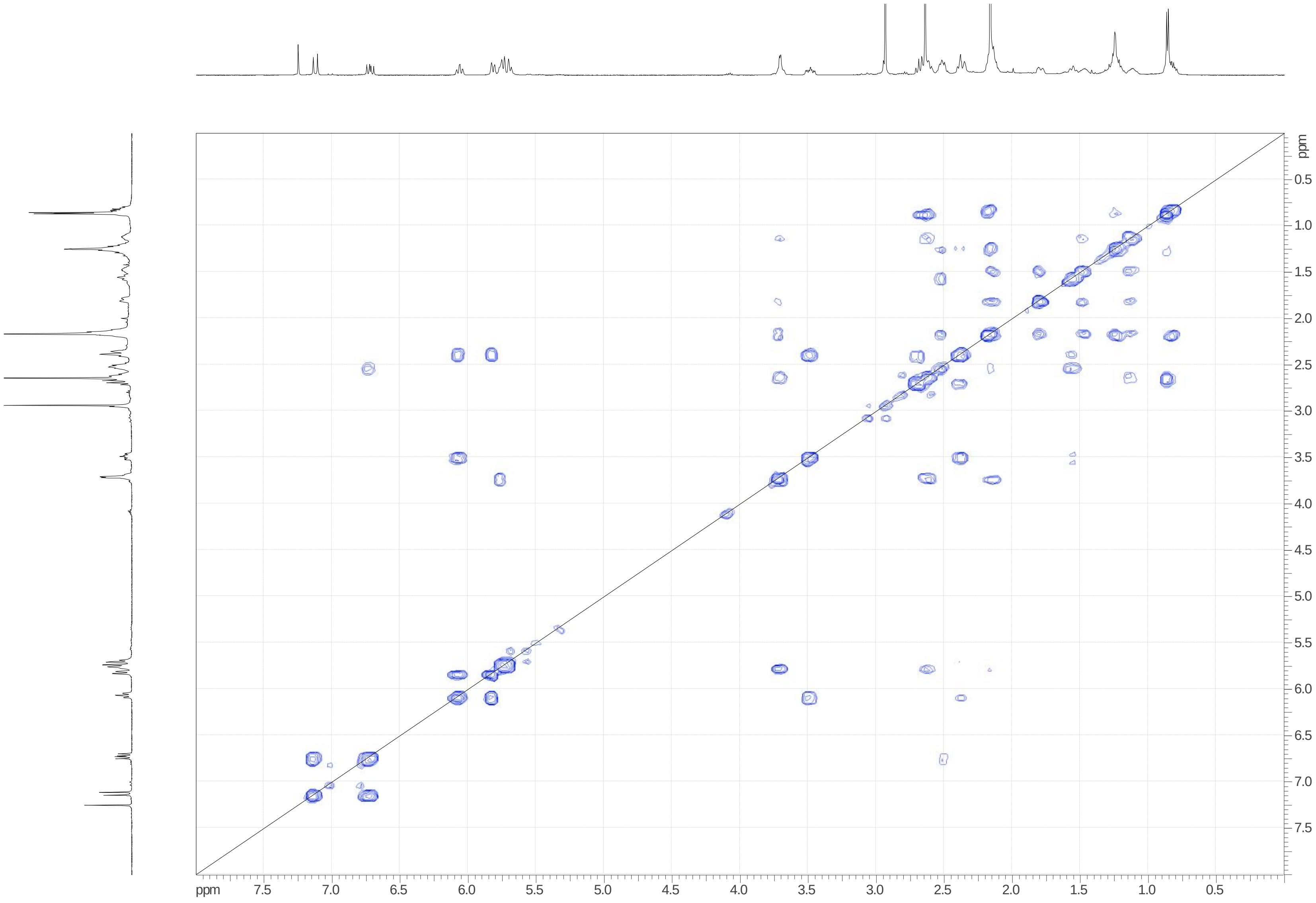

Figure S3. *Cont.*

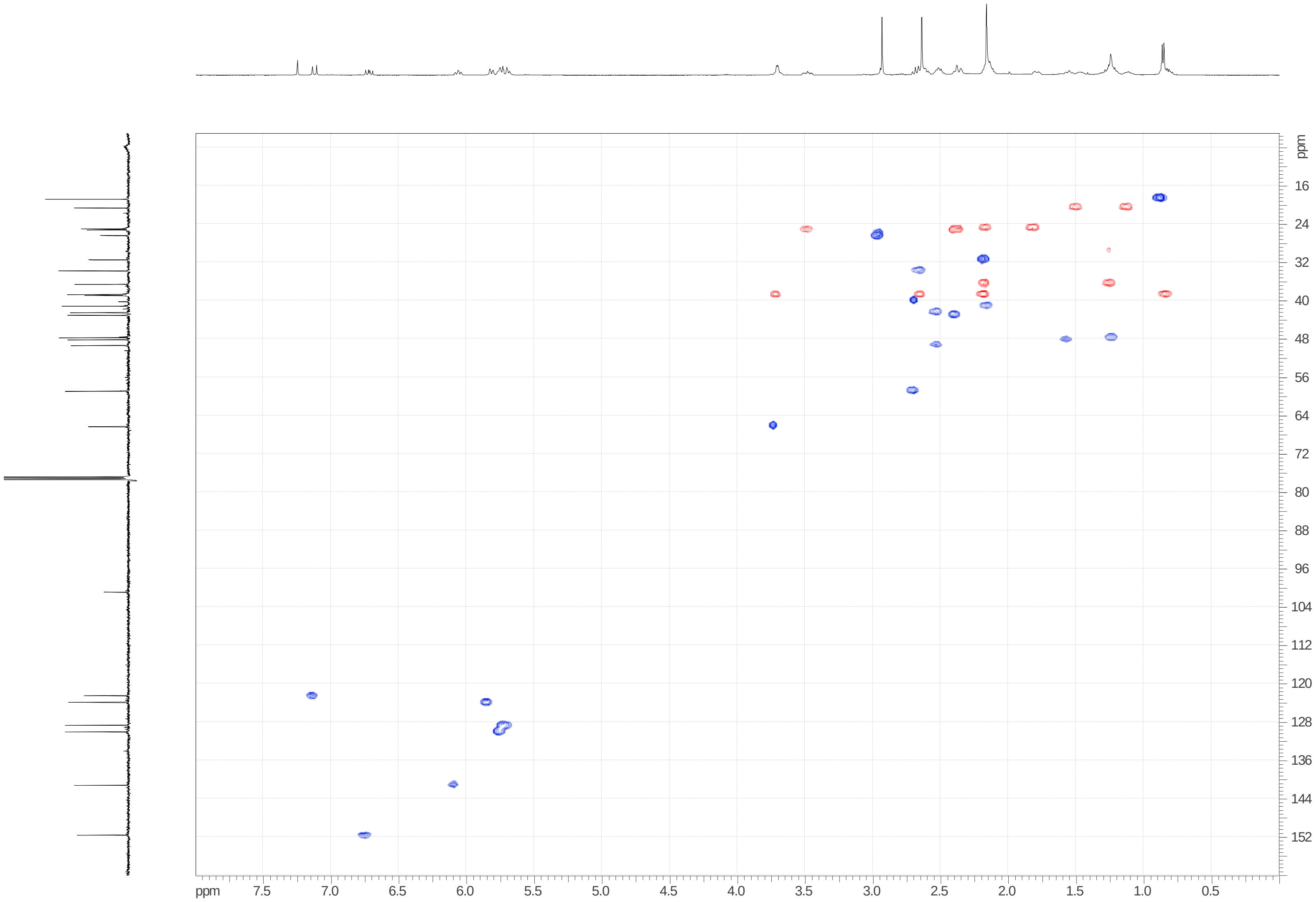

Figure S3. *Cont.*

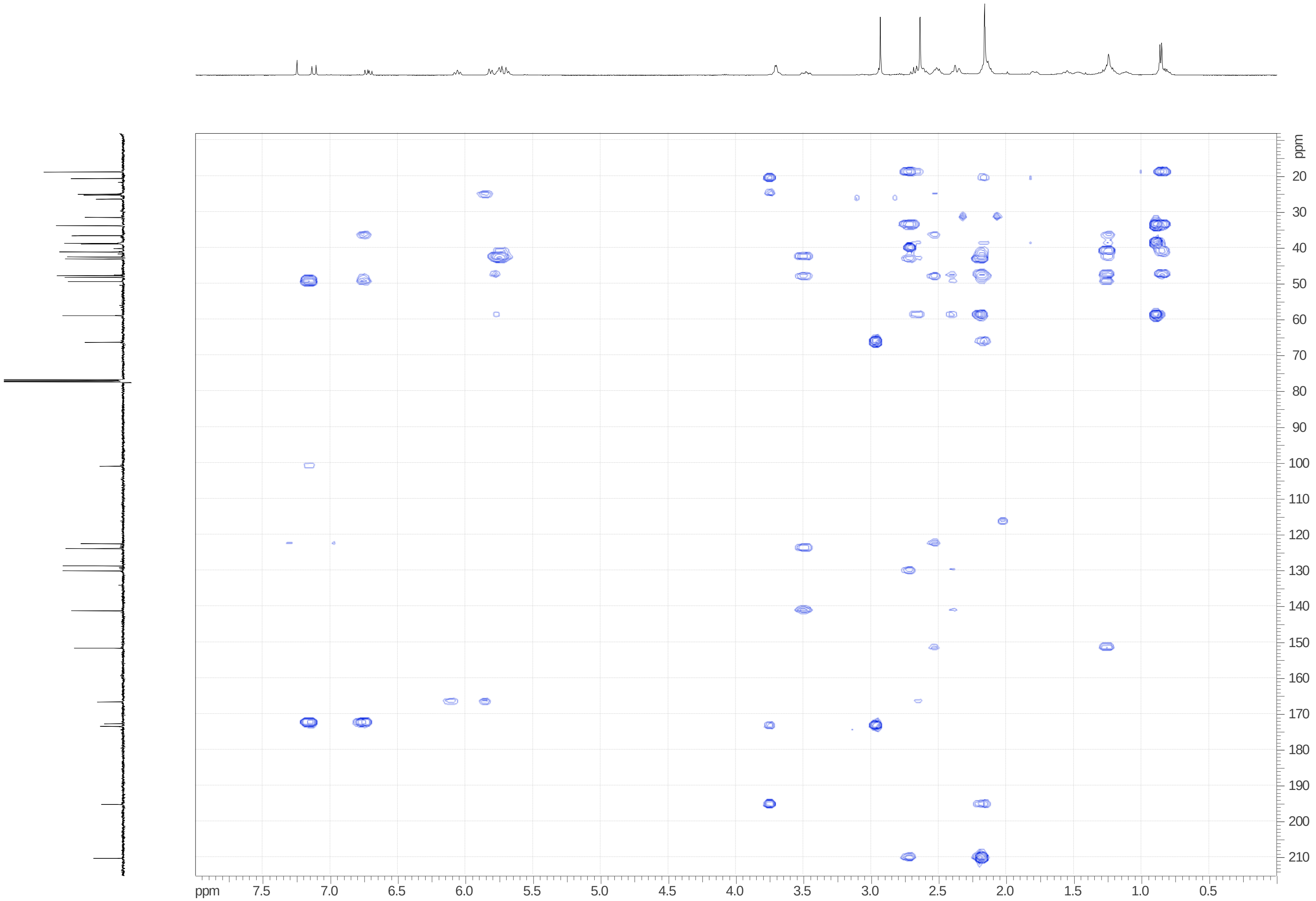

Figure S3. *Cont.*

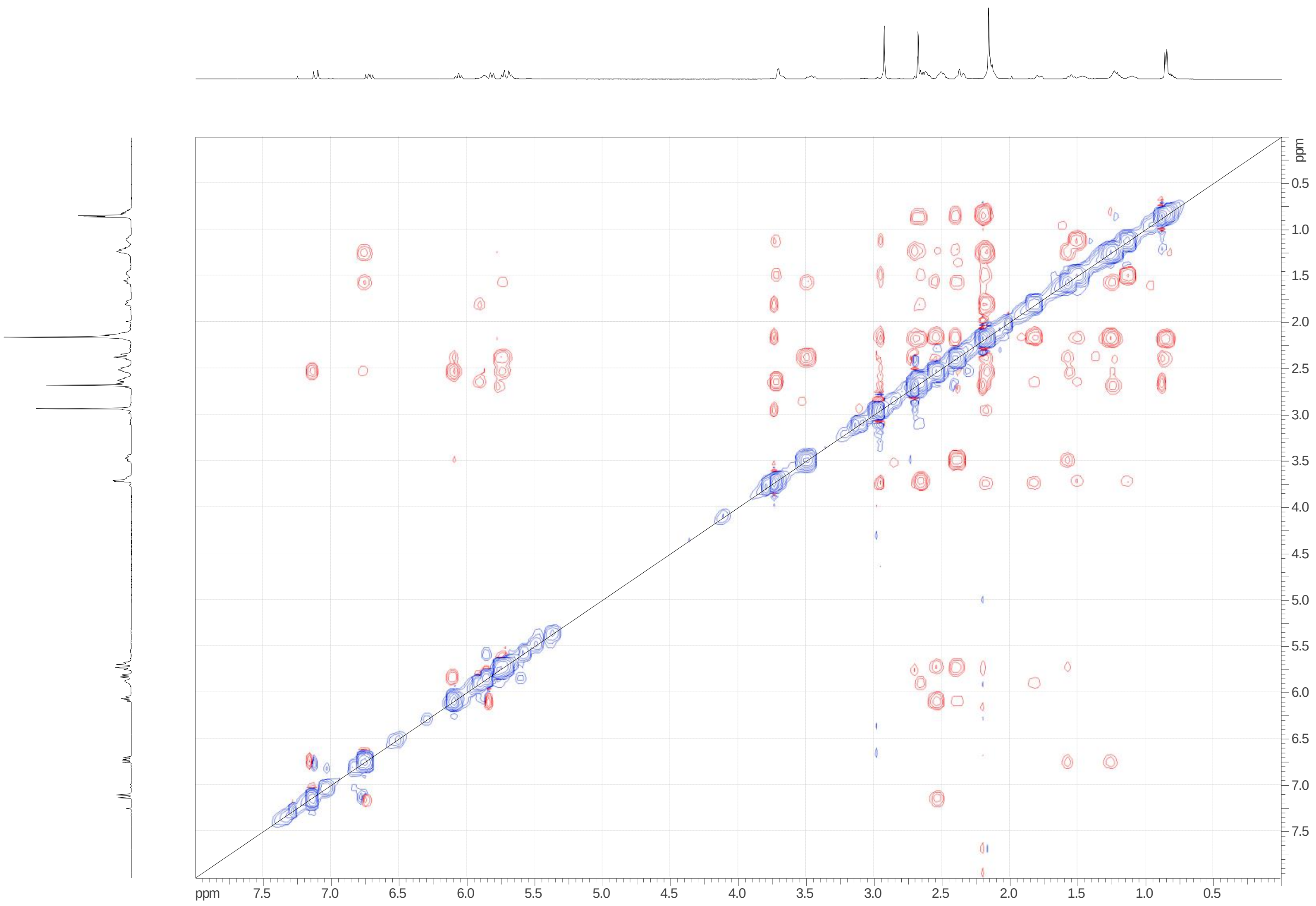

Figure S3. (**A**) ESI-TOF and UV spectra of compound **3**; (**B**) ^{1}H NMR ($CDCl_3$, 500 MHz) of compound **3**; (**C**) ^{13}C NMR ($CDCl_3$, 125 MHz) of compound **3**; (**D**) COSY of compound **3**; (**E**) HSQC of compound **3**; (**F**) HMBC of compound **3**; (**G**) NOESY of compound **3**.

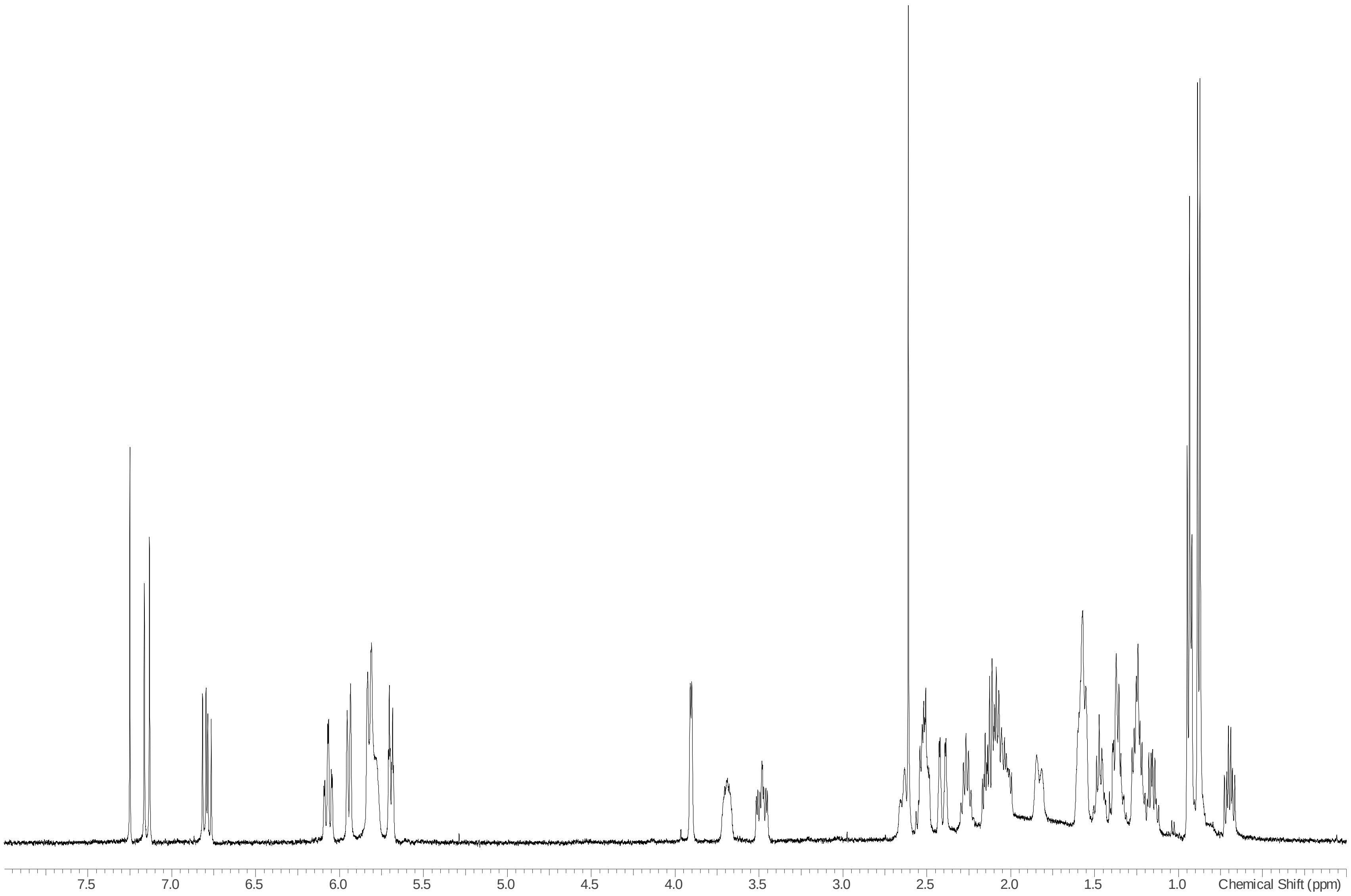

Figure S4. *Cont.*

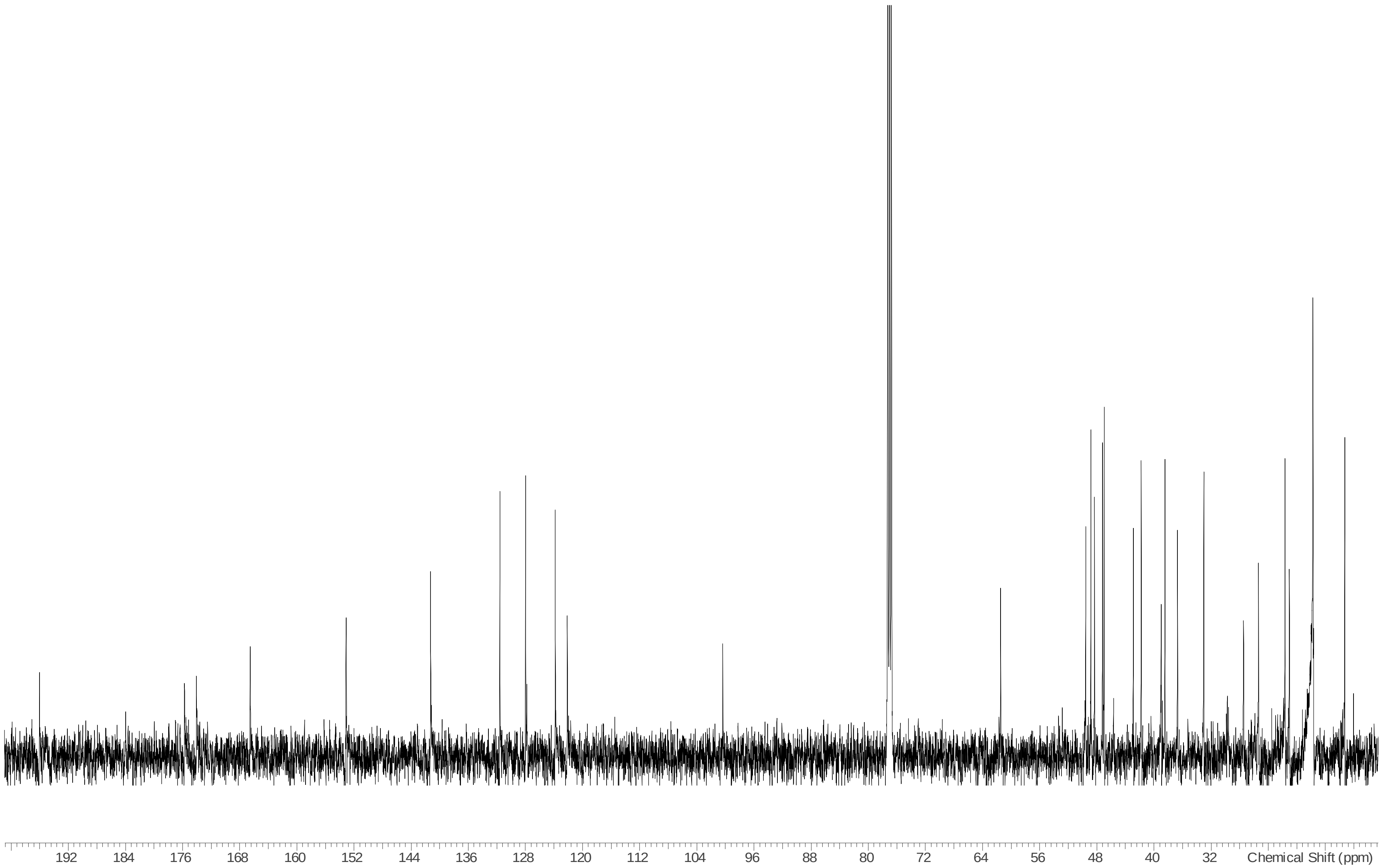

Figure S4. *Cont.*

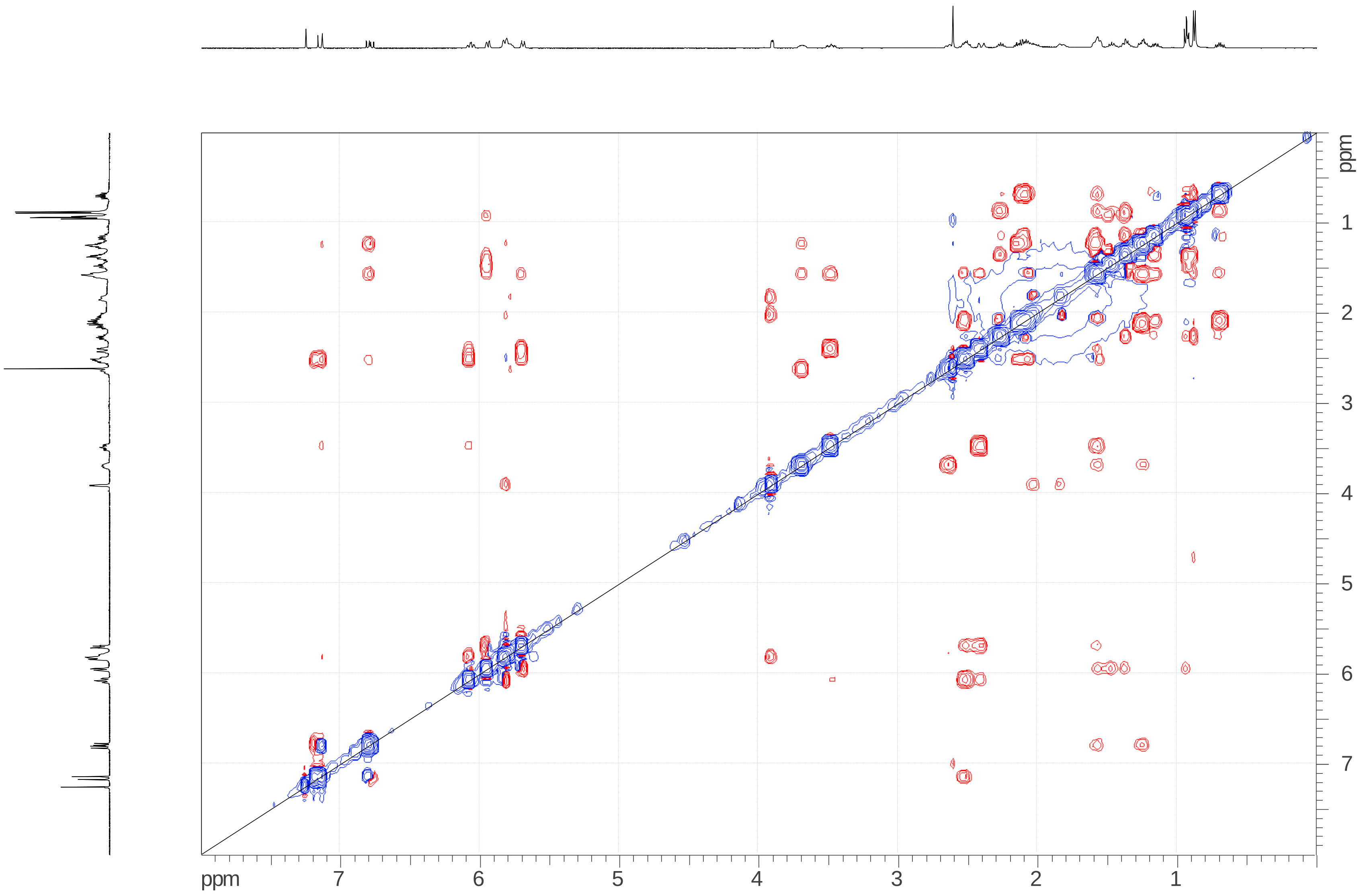

Figure S4. (**A**) ^{1}H NMR (CDCl$_3$, 500 MHz) of compound **4**; (**B**) ^{13}C NMR (CDCl$_3$, 125 MHz) of compound **4**; (**C**) NOESY of compound **4**.

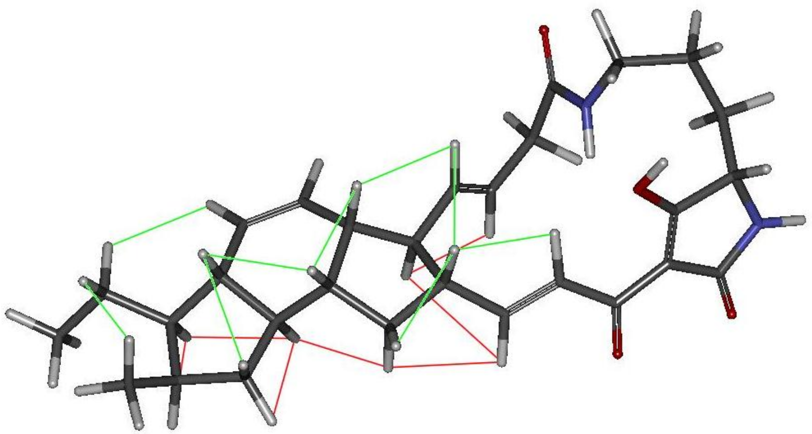

1

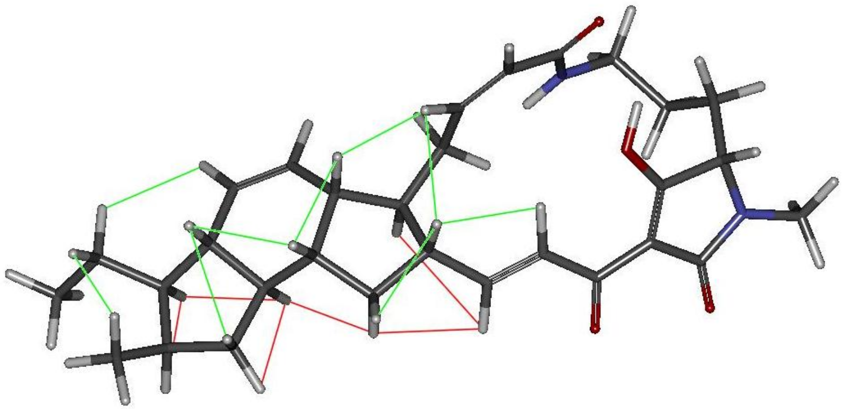

2

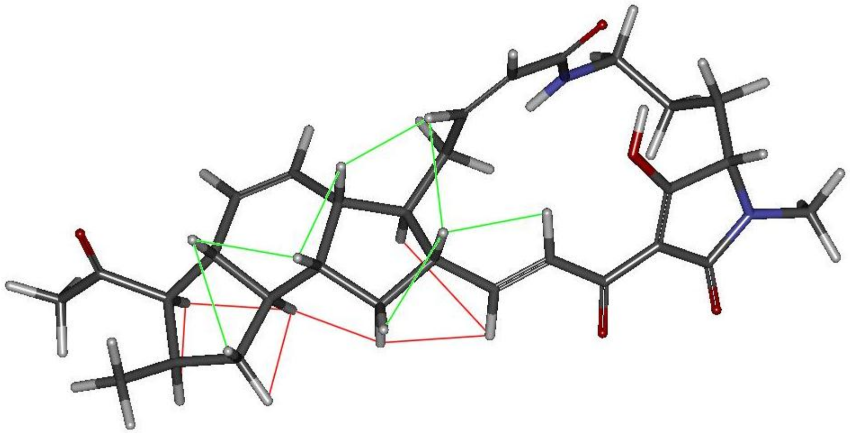

3

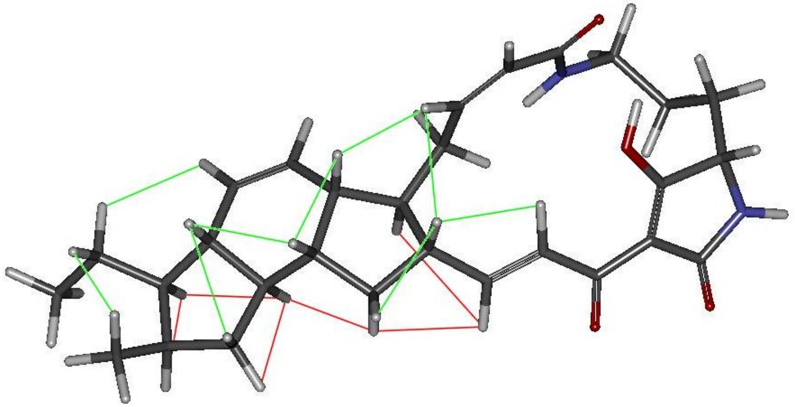

4

Figure S5. Molecular models of **1**–**4** showing the key observed NOEs which determine the relative configuration for all compounds. The protons in β orientation (relative to de fused tricycle pseudoplane according to the 2D structure scketches) which display mutual correlation are connected by green lines while red lines are employed for those in α orientation.

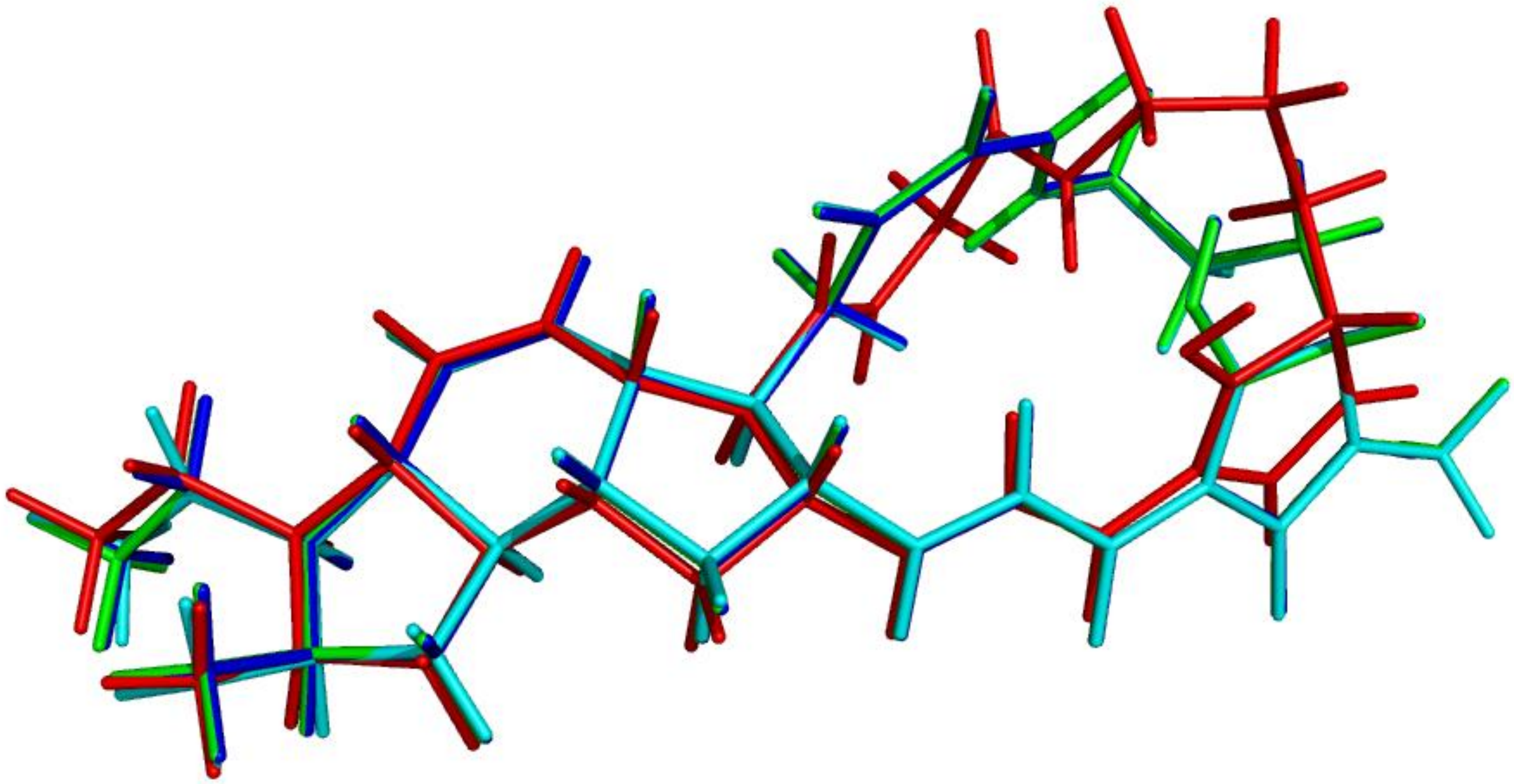

Figure S6. Overlay of the molecular models of **1**–**4**. The following colour coding was employed: Red (**1**), green (**2**), cyan (**3**), blue (**4**).

Table S1. NMR data of Ikarugamycin (**4**). ^{1}H and ^{13}C NMR (500 and 125 MHz in $CDCl_3$).

Position	δH, Mult (*J* in Hz)	δC, Mult	Position	δH, Mult (*J* in Hz)	δC, Mult
1		196.0, C	17	2.27, ddd (7.6, 7.6, 7.6)	33.0, CH
2	3.91, br s	61.5, CH	18	0.69, ddd (12.0, 12.0, 6.8) 2.11, m	38.4, CH_2
3	1.81, m; 2.09, m	27.4, CH_2	19	1.16, ddd (11.1, 11.1, 4.1)	48.8, CH
4	1.24, m; 1.57, m	21.0, CH_2	20	2.08, m	41.8, CH
5	2.64, s; 3.67, br s	38.9, CH_2	21	1.26, q (6.0, 5.1) 2.10, m	36.7, CH_2
NH-6	5.92, br s		22	2.56, dd (10.6, 6.6)	49.5, CH
7		166.5, C	23	6.80, dd (15.4, 10.6,)	153.0, CH
8	5.83, d (10.6)	123.8, CH	24	7.14, d (15.4)	122.3, CH
9	6.02, dd (10.6, 10.6)	141.3, CH	25		175.4, C
10	2.40, d (10.6); 3.46, m	25.3, CH_2	26		100.4, C
11	1.57, m	48.3, CH	27		174.1, C
12	2.50, ddd (11.5, 7.7, 3.7)	42.9, CH	NH	6.15, br s	
13	5.67, dd (10.0, 3.7)	128.1, CH	29	0.86, d (7.2)	17.7, CH_3
14	5.94, d (10.0)	131.6, CH	30	1.45, m 1.35, m	21.6, CH_2
15	1.57, m	47.0, CH	31	0.92, t (7.0)	13.3, CH_3
16	1.37, m	47.2, CH			

© 2014 by the authors; licensee MDPI, Basel, Switzerland. This article is an open access article distributed under the terms and conditions of the Creative Commons Attribution license (http://creativecommons.org/licenses/by/4.0/).
